# Aging Modulates Prefrontal Plasticity Induced by Executive Control Training

**DOI:** 10.1093/cercor/bhaa259

**Published:** 2020-09-15

**Authors:** Hugo Najberg, Laura Wachtl, Marco Anziano, Michael Mouthon, Lucas Spierer

**Affiliations:** Neurology Unit, Medicine Section, Faculty of Science and Medicine, University of Fribourg, 1700, Fribourg, Switzerland; Neurology Unit, Medicine Section, Faculty of Science and Medicine, University of Fribourg, 1700, Fribourg, Switzerland; Neurology Unit, Medicine Section, Faculty of Science and Medicine, University of Fribourg, 1700, Fribourg, Switzerland; Neurology Unit, Medicine Section, Faculty of Science and Medicine, University of Fribourg, 1700, Fribourg, Switzerland; Neurology Unit, Medicine Section, Faculty of Science and Medicine, University of Fribourg, 1700, Fribourg, Switzerland

**Keywords:** aging, ERP, inhibitory control, plasticity, training

## Abstract

While declines in inhibitory control, the capacity to suppress unwanted neurocognitive processes, represent a hallmark of healthy aging, whether this function is susceptible to training-induced plasticity in older populations remains largely unresolved. We addressed this question with a randomized controlled trial investigating the changes in behavior and electrical neuroimaging activity induced by a 3-week adaptive gamified Go/NoGo inhibitory control training (ICT). Performance improvements were accompanied by the development of more impulsive response strategies, but did not generalize to impulsivity traits nor quality of life. As compared with a 2-back working-memory training, the ICT in the older adults resulted in a purely quantitative reduction in the strength of the activity in a medial and ventrolateral prefrontal network over the 400 ms P3 inhibition-related event-related potentials component. However, as compared with young adults, the ICT induced distinct configurational modifications in older adults’ 200 ms N2 conflict monitoring medial–frontal functional network. Hence, while older populations show preserved capacities for training-induced plasticity in executive control, aging interacts with the underlying plastic brain mechanisms. Training improves the efficiency of the inhibition process in older adults, but its effects differ from those in young adults at the level of the coping with inhibition demands.

## Introduction

Inhibitory control (IC) refers to the capacity to inhibit motor or cognitive processes. This executive component is notably involved in suppressing impulsive or habitual responses, as typically required for successful goal-directed behaviors (e.g., Logan et al. 1997). Because IC relies largely on ventrolateral prefrontal—striatal brain structures that rapidly deteriorate with aging (Aron et al. 2014), declines in IC performance can already be observed in 60–70 years old populations (e.g., Nielson et al. 2002).

Training IC by the repeated practice of inhibition tasks has thus been advanced as a potential approach to compensate the executive deficits associated with healthy aging (e.g., Anguera et al. 2013; Heinzel et al. 2014). Yet, whether IC is actually susceptible to improvement with training in older adults remains largely unresolved. Moreover, the neural mechanisms supporting IC plasticity in older populations, and how they differ from those in young populations is also underexplored.

Less than five studies investigated the brain functional or structural correlates of plasticity induced by inhibitory control training (ICT) in older adults. They observed improvements at the trained inhibition tasks (Mozolic et al. 2010; Ji et al. 2016; Kühn et al. 2017), associated with increases in right inferior frontal gyrus (rIFG) activity at rest (Mozolic et al. 2010), inhibition-related left ventrolateral prefrontal cortex activity (Hartmann et al. 2019), and rIFG cortical thickness (Kühn et al. 2017; Nguyen et al. 2019 for a review). Although scarce, these results suggest different effects of ICT in older and young populations, the latter typically showing decreases in rIFG activity with training (Manuel et al. 2013; Chavan et al. 2015; Hartmann et al. 2016).

In the present study, we investigated the spatio-temporal brain mechanisms underlying training-induced changes in IC in older adults. We also analyzed how these plastic changes differ from those in young populations. We trained young (18–40 years old) and older (60–75 years old) healthy participants for 3 weeks on a Go/NoGo task (one group of 29 older participants and one group of 32 young) and on a control 2-back task (one group of 28 older participants). The training tasks were gamified and completed from home to increase the engagement and the adherence to the intervention (Mishra et al. 2016). They included autoadaptive difficulty levels to maintain the tasks challenging and to control that changes in difficulty did not confound between-groups and between-sessions comparisons.

The effects of the training were examined with behavioral measures and event-related potentials (ERP) recorded before and after the intervention during a Go/NoGo task similar to the trained task. To test the generalization of the effect of the ICT, we recorded participants’ self-reported quality of life (QoL) and impulsivity before and after the training interventions. A key aim of cognitive training intervention in older population is indeed to help recovering the deficits influencing QoL and we thus focused on this real-life oriented endpoint (Beckert et al. 2012; Rabelo Pereira et al. 2015), together with a lower-level personality impulsivity trait also putatively influenced by IC performance (Roberts et al. 2011).

We analyzed ERPs in the so-called “electrical neuroimaging” framework, an approach combining data-driven, time-frame-wise robust statistical analyses of global descriptors of the scalp field potentials distribution (i.e., the global field power, GFP, and the topography of the ERPs) and source estimations analyses. This approach enables deeper neurophysiological interpretations than local analyses of single electrodes’ amplitude of ERP components: Since differences in ERP topographies necessarily follow from changes in the configuration of the underlying neural generators (Lehmann, 1987), and GFP modulations follow from changes in the response strength of the generators (Murray et al. 2008; Michel and Murray 2012; Tzovara et al. 2012), the pattern of modulations in these two metrics can be interpreted mechanistically. A change in GFP without change in topography indicates a purely quantitative variation in the response gain of identical configurations of brain sources. Such strength-based plastic mechanisms as typically associated with changes in processing efficiency. In contrast, a change in ERP topography (with or without GFP modulations) indicates qualitative modulations of the generators’ configuration. Such network-based plastic mechanisms typically index the development of new response strategy or compensatory activity (Kelly and Garavan 2005).

As a second step, distributed source estimations are computed and statistically analyzed with the same designs as the ERPs to localize in the brain the origin of the effects observed at the scalp level (Grave De Peralta Menendez et al. 2004).

We first examined the effect of the ICT in older adults based on the interaction term of a 2*2 mixed design with Session (pre-; post-training) as within-subject factor and Training (Go/NoGo training; control 2-back training) as between-subject factor applied to the performance and to electrical neuroimaging activity during the inhibition trials of a Go/NoGo task. Using a 2-back task training as an active control allowed to rule out that the observed effects were due to retest, participating in an intervention, practicing an executive task, or being repeatedly exposed to the trained stimuli.

We predicted that as compared with the active control group, the Go/NoGo training group would show larger improvements in inhibition performance. Functionally, this group may either (1) show larger improvement in the efficiency of the underlying brain networks and thus quantitative decreases in brain electrical activity indexed by decreases in the GFP without concomitant topographic modulations or (2) develop compensatory strategy by recruiting additional areas, which would be indexed by topographic modulations. These changes should manifest during the two key IC processing phases: the 250–350 period of the N2 component indexing conflict monitoring (Nieuwenhuis et al. 2003; Donkers and Van Boxtel 2004; Schmajuk et al. 2006; Enriquez-Geppert et al. 2010; Gajewski and Falkenstein 2013) and the 350–500 ms period of the P3 inhibition implementation component (Smith et al. 2008; Albert et al. 2013; Gajewski and Falkenstein 2013). We finally expected these ERP modulations to be respectively driven by changes in anterior cingulate, presupplementary motor area (pre-SMA), and bilateral ventrolateral prefrontal cortex (VLPFC) activity (Spierer et al. 2013 for review). The “improved efficiency” model (1) would predict a decrease in activity in these areas, whereas the “compensatory model” (2) would predict an increase in activity in these areas.

Regarding generalization patterns, we predicted an absence of transfer to QoL and impulsivity traits, given that very limited transfers of ICT or of other types of executive training have been observed (e.g., Simonet et al. 2019); recent meta-analyses indeed indicate that training generalization, if any, only manifests in very close tasks (e.g., Sala and Gobet 2019).

We then tested the hypothesis that aging would interact with the effects of the ICT. The baseline differences in IC performance and in functional IC organization between young and older populations should indeed result in qualitatively different effects of the training; prefrontal structural deteriorations may indeed not only modify older adults’ capacity for plastic reorganizations, but also result in the engagement of compensatory functional activity not present in young adults. Hence, different networks being initially engaged by the two groups in the tasks, they should show different patterns of modifications with training (Park and Bischof 2013 for discussion).

We tested this hypothesis based on the interaction term of a 2*2 Session by Age (young; older adults) mixed design, applied to the same dependent variables as for the first contrast.

We predicted that the older group would show larger improvements than the young group at the Go/NoGo task because their initial deficit would leave more room for improvement (slower response times; e.g., Nielson et al. 2002; Vallesi et al. 2011; Heilbronner and Münte 2013; Hong et al. 2014; and more inhibition failures; e.g., Nielson et al. 2002; Langenecker and Nielson 2003). At the electrophysiological level, we predicted different network configuration changes between the two groups, that should manifest as topographic ERP modulations during the N2/P3 ERP components, driven by a larger decrease in activity in the areas typically exhibiting additional compensatory functional activity in the older adults, that is, the anterior cingulate and pre-SMA/bilateral VLPFC (Cabeza et al. 2002; Nyberg et al. 2003; Cappell et al. 2010; Mozolic et al. 2010; Hsieh and Fang 2012; Anguera et al. 2013; Heinzel et al. 2014; Coxon et al. 2016). This decrease would possibly index a return to a network configuration more similar to that of the young-like physiological state. As for the first contrast, we also posited that the training may reinforce compensatory strategies in the older group, which would manifest as an increase in the activity in these areas over the same time period (Sebastian et al. 2013; Reuter-Lorenz and Park 2014).

Regarding putative difference in generalization patterns between the two groups, directional predictions are difficult to formulate. There is indeed very few literature on how generalization interacts with aging. We however expected an absence of generalization between the two groups (e.g., Sala and Gobet 2019).

## Materials and Methods

### Participants

The participants were recruited via advertisement at the University of Fribourg and at organizations working with older adults. Participants were compensated for their participation with the tablet used for the intervention. All experimental protocols were approved by our local ethics committee, protocol #2017-01889. The experimental sessions were undertaken with the understanding and written consent of each participant.

Inclusion criteria were: signed informed consent; right-handedness; 18–40 years old for the Young group; and 60–75 years old for the older group. Exclusion criteria were: history of diagnosed neurological or psychiatric disorders, and for the older group only: the Montreal Cognitive Assessment (MoCA; Nasreddine et al. 2005) score below 26/30 and the Frontal Assessment Battery (FAB; Dubois et al. 2000) score below 15/18 to rule out any abnormal neurocognitive impairment (see Table 1 for detailed information).

**Table 1:** Demographic data and neuropsychological tests results

Mean ± SD	Young Go/NoGo training (*n* = 32)	Older Go/NoGo training (*n* = 29)	Older 2-back training (*n* = 28)
Age	23.73 ± 3.29	66.72 ± 4.05	67.45 ± 3.93
Gender ratio (M/F)	0.47	0.39	0.38
FAB (/18)	-	17.07 ± 0.88	17.24 ± 1.06
MoCA (/30)	-	27.72 ± 1.62	27.31 ± 1.61

Our sample size was determined a priori with a power calculation based on previous studies by our group on IC training and plasticity, which suggest that medium effect sizes could be expected on the key behavioral and functional measures in the present study (Manuel et al. 2010; Enge et al. 2014; Chavan et al. 2015; Hartmann et al. 2016; Kühn et al. 2017). For rmANOVA, targeting a within-between two by two interaction as in the present study, a power of 0.8, and a medium effect size *f* = 0.2 (0.05 α threshold), G-Power indicates that a total sample size of sample of *n* = 26 is necessary (Erdfelder et al. 2009). Yet, because we focused on an older adult population potentially showing different effect of training than the young population on which most of previous ICT studies were conducted, and that effect size may have been overinflated in previous literature, we planned a sample size of approximately 30 per group.

A total of 91 participants were finally recruited for the study: Two older participants were excluded due to a failed MOCA and one for consent withdrawal. This left 32 young and 29 older participants for the Go/NoGo intervention groups, and 28 older participants for the 2-back intervention group. Of note, the young group mean age was in the lower range of the 18–40 year age-related inclusion criteria planned for this group because the recruitment on the University campus was successful and we did not have to recruit a larger (and possibly older) population.

### Experimental Procedure

Data were collected during two sessions in the EEG laboratory of the Neurology Unit of the University of Fribourg and during the intervention by an automatic upload from the tablets to our server.

Older participants were randomly assigned to either the experimental Go/NoGo training or to the control 2-back training. A different laboratory member conducted the pre- and the post-training session, allowing to blind the experimenter of the post-training session to the training group of the participant and thus to control that experimenters’ expectations on the effect of the training did not confound our results.

During the first session, participants were first instructed to read the informed consent form and to sign it if they agreed to participate. The French version of the FAB and MoCA questionnaires were then administered to older participants. All participants then filled a custom General Health Questionnaire (GHQ) to screen for the inclusion/exclusion criteria, a custom QoL questionnaire, and the French-translated version of the Barratt Impulsiveness Scale (BIS-11; Patton et al. 1995).

Then, the experimenter installed the EEG system, and participants completed a Go/NoGo task (6 blocks of 60 trials), a two-back task and a Flanker task. Ten minutes breaks were proposed between each task. When finished, the EEG was removed, and the participants were instructed on the home-based training intervention and given the tablet with the application. Of note the experimenter explaining the training task was informed about the condition assignment only after the end of the pretraining session, just before instructing the participant about the training tasks. Another experimenter blind to the condition assignment was in charge of the post-training recording. At the end of the pretraining session, participants received both written and oral instructions on their training task. They were informed that they had to practice the task they were assigned to, but they were unaware of the existence of another group performing a different task.

For the Go/NoGo training, participants were instructed to drag and drop the food item as fast as possible only when it belonged to a target category. For the 2-back training, participants were instructed to drag and drop the item only if the penultimate item belonged to the same category (see the Task section for details). All participants were instructed that the goal was to reach the highest score they could at each block.

After the 3 weeks of training intervention (5 days with 20 min training per week), participants came back to the lab. They filled three questionnaires: a custom debriefing questionnaire, and the same BIS-11 and QoL questionnaire. Next, they completed the same EEG Go/NoGo task as before the training, but with a new randomized block order.

### Questionnaires

The participants in the older and/or young adult group completed or were administered the following questionnaires: (1) *MOCA*: a one-page 30-point test measuring general cognitive functions with tests of visuospatial abilities, language, attention, short-term memory, and temporal orientation; (2) the *FAB*: a short test of frontal efficiency assessing conceptualization, mental flexibility, motor programming, sensitivity to interference, IC, and environmental autonomy; (3) the *BIS-11*: 30 items on a 1-to-4 scale on the frequency of impulsive behavior; (4) the *GHQ*: a custom-made questionnaire on sleep and food, caffeine, cigarettes and alcohol consumption at the day of the session, self-report of height and weight, current or past health problems (surgery, medications, visual and auditory acuity, etc.), and pregnancy; (5) *the QoL*: 10 items on a 1-to-10 scale on participants’ perceived capacity to concentrate and inhibit, self-control, and every-day life satisfaction (e.g., “On a scale between 1 (‘Not at all’) to 10 (‘Completely’): Are you satisfied with your ability to concentrate? ”); and (6) a *Debriefing questionnaire:* six questions on the everyday life use of digital technology and feeling/perception of the intervention.

### Stimuli

The stimuli in the training task and in the pre- and post-training EEG tasks were pictures selected from the Food-Pics database (Blechert et al. 2014) and divided into 10 categories: meat, sandwiches, chocolate, bread, fruits, vegetables, chocolate cake, fruit, cake, and cheese.

### Pre- and Post-training Session

We used the E-Prime 3.0 software (Psychology Software Tools, Inc.) for stimulus presentation and response recording.

#### Go/NoGo Task

Participants were instructed to respond as fast as possible to a specific category of stimuli (Go) by pressing a button on a response box with their right index finger, while withholding their responses to another category of stimuli (NoGo). A total of 6 blocks of 60 trials were completed by each participant, separated by 2 min breaks. Each block consisted of 36 Go and 24 NoGo trials presented randomly. The Go and NoGo categories were pseudo-randomly chosen across participants, so that the same NoGo and Go categories were never used twice and the order of the Go and NoGo used across blocks were different from the two sessions of the participant.

Before the beginning of each block, participants were presented with spoken and written instructions about the Go and NoGo stimuli category. During the blocks, the median response time (RT) was continuously adjusted as the median of the previous correct Go trials to compute a response time threshold (RTT). Accordingly, the initial RTT value corresponded to the RT of the first correct Go trial and then dynamically changed depending on the participant’s performance at each trial of the block (median of RTs for correct Go). The RTT was then used to provide feedback on response speed to the Go trials. This procedure enabled to maintain the same level of time pressure across participants and blocks, that is, independently of any initial interindividual differences in Go/NoGo performance and of change in performance with the intervention (for corresponding procedures, see e.g., Manuel et al. 2010; De Pretto et al. 2019). The feedback on RT thus increased the tendency to respond when a stimulus was presented, and in turn the need for inhibition during NoGo trials.

**Figure 1:**
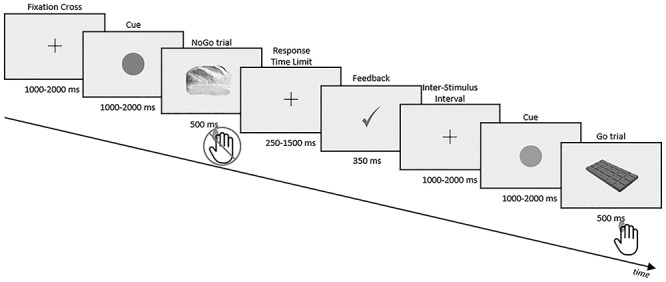
Timing of the Go/NoGo task.

Each experimental trial consisted in the sequential presentation of (Fig. 1):

- a black fixation cross on a gray screen with a random duration between 1000 and 2000 ms,
- a red or green circle (randomly between 1000 and 2000 ms),
- the stimulus (500 ms) with a response window terminating as soon as the participant responded, but with a minimal-maximal duration of 250–1500 ms,
- a feedback on the performance (350 ms) that could be either: a green check mark after fast Hit trials (response after a Go stimulus, RT < RTT) or correct rejections (CR, no response after a NoGo stimulus), an orange feedback “Too late!” after hits with a RT > RTT, or a red cross after misses (no response after a Go stimulus) or false alarms (FA; response after a NoGo trial).

The participants were instructed that a stimulus preceded by a green cue had a high chance of being a Go trial and the opposite if preceded by a red cue. After a green cue, Go trials had a 70% chance of appearing, and a 30% chance to appear after a red cue. This parameter was implemented for gamification purposes and was not considered in the present analyses. We still verified that the mixing of both cue types in our analyses had no impact on the results (see Supplementary Material).

We also recorded our participants’ performance during a 2-back and a Flanker task before and after the training (see Supplementary Material). The data from the Flanker task could not be used because due to a technical problem we could not collect enough data to run reliable analyses. The data of the 2-back task will be the focus of a future study.

### Intervention

The experimental Go/NoGo and the control 2-back interventions were implemented as android applications developed on the 2018 version of Unity (Unity3d.com 2015). The two software are available upon request to the authors.

Participants had to play four training sessions of 3 blocks (1′45 each) per day, 5 days a week, for 3 weeks (total of 60*5′15 practice time). In each block, participants were presented with food pictures and instructed to drag the targeted items toward the bottom of the screen. All experimental parameters (timing, pictures, stimuli categorization, probability of targeted item, and RTT rule) were the same as in the pre-/post-training tasks. Cues on the probability of the forthcoming item type were given as green, orange, or red cue before every stimulus. Go items were likely to be presented after green cues (70%); less likely after orange cues (50%); and unlikely after red cues (30%).

We designed the intervention tasks following well-established video-game design principles to create a satisfying experience, reinforcing intrinsic motivation, and in turn engagement and adherence (Hunicke et al. 2004; Schell 2008). To this aim, we included a risk–reward system in which participants had to validate a jackpot after successive correct responses to win points. The more correct responses they accumulated in a row, the larger the jackpot got. However, after an error, the jackpot reset to zero, and the participant lost all uncashed points. In the application starting screen, we included a progression histogram displaying the evolution of the participants’ response time, accuracy, and score at each training day. For young participants, a peer-ranking system and a score goal was introduced and displayed after every session.

To reinforce the motivation to improve performance, feedbacks were given on the participant’s performance based on their score after every session: the higher was their score, the happier was the character in the dialog box. In addition, before starting a block, an advice was given on response strategy based on their previous speed/accuracy response pattern (“Try to be quicker!,” “Too many mistakes!” , “Pay attention to the jackpot,” “Congratulations, continue like that”). This procedure was implemented to minimize potential drifts toward more cautious or more impulsive response mode with training. We then provided a reminder on the task instruction and of the target “Go” category. Visual and auditory arts were used to improve the immersion, the experience of the participants and to support the positive and negative feedback on performance.

### Behavioral Data Recording and Pre-processing

#### Behavioral Dependent Variables

We recorded the RT to the Go stimuli (correct responses, or “Hit” trials), as well as the rate of commission errors (incorrect responses to NoGo stimuli, or “false alarms,” FA), and of omission errors (no response to Go stimuli, or “Miss”).

The performance was assessed based on the mean RT of Hit trials and on the mean FA rate.

#### Behavioral Data Pre-processing

For the data collected during the pre- and post-training sessions, we first excluded hit and FA trials with a RT below 100 ms, since these responses were forcibly unrelated to the presented stimulus. Then, we removed blocks with response pattern indexing a disengagement from the task or misunderstanding of the instructions (such as above chance error rates, etc.) or performance outside participant’s “normal” pattern. Exclusion criterion were thus defined on principled grounds or based on distributions parameters as follows:

- Blocks with FA rate above 0.7; miss rate above 0.2; FA rate above the participant’s intrasession median + 0.2; miss rate above the participant’s intrasession median + 0.1.

If more than half of the blocks in a given participant’s experimental session were excluded, the participant’s whole data were removed for that task.

Finally, hit trials with RT outside the 2.5*standard deviation upper and lower thresholds around the mean were excluded for each participant and each session.

For data collected during the intervention, only hit and FA trials with a RT below 100 ms were excluded before running the analyses.

#### Statistical Approaches

For all our behavioral analyses, we set our statistical threshold at alpha = 0.05 and used Holm-Bonferroni corrections for multiple tests when necessary. We also choose to report the following standardized effect sizes: *r* for *t*-tests and correlations, and η_G_^2^ for mixed ANOVAs. 2*2 interactions were further explored using pre- versus post-training dependent sample *t*-tests.

Given the limitation of the frequentist approach to provide support for the null hypothesis, we further investigated the nonsignificant 2*2 interactions with Bayes factors (BF) analyses using the R package BF (Morey et al. 2018) with default parameters (i.e., *r* scale fixed effects = 0.5; *r* scale random effects = 1; number of iterations = 10 000). The probability of the data supporting an absence of interaction (H0) is computed by dividing the BF of the interaction model against the BF of the full model without interaction, resulting in a BF_01_, where BF_01_ > 3 (i.e., H0 three times more likely than H1) is set as the significant threshold (Dienes 2011).

**Table 2:** Average number of trials and rejected epochs

Mean ± SD	Young Go/NoGo training	Older Go/NoGo training	Older 2-back training
CR trials	99.8 ± 22.1	106.4 ± 17.9	110.3 ± 16.1
Rejected CR epochs	2.8 ± 7.6	1.7 ± 4.5	3.3 ± 6.8

#### Behavioral Data Analyses

If not otherwise specified, analyses were computed using the basic R functions. The 2*2 ANOVAs and their effect sizes were computed using the “ez” R-package (Lawrence 2016).

### Sanity Checks

As a sanity check for the effect of task practice, we expected the participants’ performance in the training task to improve during the training. This was tested by applying a linear model of the two mean RT hit and mean FA rate Go/NoGo dependent variables on the factor training days (day 1–15), computed separately for the young and older group. Additionally, the differences between the young and older models were assessed using the interaction term of a group (older vs. young) × days generalized linear model (GLM) and reported in the Supplementary Material.

As a sanity check for the sensitivity of our task to aging, we examined if the older group indeed showed lower performance than the young group at the pretraining Go/NoGo using independent sample *t*-tests.

As criteria to ensure a loading of our Go/NoGo tasks on IC, mean RT Hits around 400 ms and FA rates around 10–15% were expected to ensure response prepotency and the involvement of inhibition. These values are based on previous studies with similar tasks and on the reasoning that they index speeded response and a difficult inhibition (e.g., Hartmann et al. 2016, 2019; De Pretto et al. 2019).

We first tested whether ICT in older adults would improve IC by focusing on the interaction term of a 2*2 mixed design with session (pre-; post-training) as within-subject factor and training (Go/NoGo training; control 2-back training) as between-subject factor applied to each of the behavioral dependent variables (Hypothesis 1: The effect of the Go/NoGo versus 2-back training in older adults.).

The generalization patterns of the Go/NoGo training was assessed by applying the 2*2 session by training mixed design on our questionnaires on impulsivity and QoL to test for the transfer of the effects of training on these constructs.

Then, we tested whether age interacted with ICT by focusing on the interaction term of a 2*2 session by age (young; older adults) mixed design, again applied to each of the behavioral dependent variables (Hypothesis 2: The effect of the Go/NoGo training in the older versus young adults.).

The effect of age on the generalization patterns of the Go/NoGo training was assessed by applying the 2*2 session by age mixed design on our questionnaires on impulsivity and QoL to test for the effect of age on the transfer of the effects of training on these constructs.

### E‌EG Data Recording and Pre-processing

#### E‌EG Data Recording

The 64-channel electroencephalogram was recorded at a sampling rate of 1024 Hz with a Biosemi ActiveTwo system referenced to the common mode sense-driven right leg (CMS-DRL) ground placed on each side of the POz electrode. This circuitry consists of a feedback loop driving the average potential across the montage as close as possible to the amplifier zero (cf. the Biosemi website for a diagram). For the ERP analyses, offline analyses were performed with the MATLAB-based EEGLab toolbox (Delorme and Makeig 2004) and the Cartool software (Brunet et al. 2011). Statistical analyses were performed with the free toolboxes RAGU (Koenig et al. 2011) and STEN (http://doi.org/10.5281/zenodo.1164038).

#### ERP Pre-processing

We first referenced the raw data to Cz electrode and applied band-pass filtering between 0.5 and 40 Hz. Then, sinusoidal artifacts (e.g., AC power line fluctuations) and nonstationary signals were removed on the continuous data with the EEGLab plugin CleanLine at 50 and 100 Hz (https://www.nitrc.org/projects/cleanline) and artifact subspace reconstruction, respectively (ASR, with settings recommended in Mullen et al. 2015; Chang et al. 2018).

Then, EEG epochs were segmented 100 ms pre- to 700 ms post-stimulus onset and baseline corrected on the whole epochs to correct for any remaining signal drifts. The signal was then further tested for artifacts by excluding epochs with timeframe (TF) to TF jumps of more than 30 μV in at least one electrode. We also excluded epochs with at least one TF with a voltage larger than 80 μV in at least one electrode (Table 2). All data excluded in the behavioral data preprocessing (see section above) were also excluded for the EEG analyses, except the Hit trials above the 2.5 SD threshold.

Epochs were then averaged for each participant for the CR trials of the Go/NoGo task. Once averaged, the ERPs were rereferenced to the common average reference.

Finally, we visually identified bad channel(s) in the averaged ERPs and interpolated them using multiquadric interpolation relying on radial basis functions (see Jäger et al. 2016; Jäger and Buhmann 2018). An average of 1.4 electrodes (SD = 1.5) was interpolated for the young group, 1.3 (SD = 1.5) for the older GNG training group, and 1.5 (SD = 1.5) for the older 2-back training group.

### ERP Statistical Analyses

#### General Event-Related Potentials Analytical Strategy

We conducted global analyses of the ERP focusing on the power and spatial distribution (i.e., the topography) of the whole electric field at the scalp. As compared with classical analyses of local electrode amplitude and latency, global analyses of the field potentials have the advantage of being independent on the choice of the reference electrode. In addition, they enable to differentiate effects due to modulations in the strength of the responses of statistically indistinguishable brain generators (i.e., modulations in GFP but not topography) from alterations in the configuration of these generators (i.e., modulations of the topography of the electric field at the scalp; see e.g., Michel and Murray 2012; Tzovara et al. 2012 for extensive details on this approach). Since a change in voltage amplitude can either follow from changes in the strength and/or in the topography of the field potential, local analyses can indeed not disentangle between the two different underlying neurophysiological mechanisms, and thus have a limited interpretability.

#### Locking of the ERP to N2 Component Onset

Because of the typical delay in the early ERP components induced by aging, we had to realign temporally the onset of our later N2 and P3 component of interest between the two group to allow for their comparison with the age × session contrast. To this aim, we locked the ERP of the two groups to the onset of the N2 before the statistical ERP analyses: For each condition, we identified the TF used to lock the averaged ERPs using a topographic temporal segmentation approach (Supplementary Fig. 1). We submitted the group-averaged ERP data of the young and the older adults to hierarchical clustering based on an atomize and agglomerate analysis to identify the latencies of the N2 and P3 components (Murray et al. 2008; Brunet et al. 2011). This approach is based on evidence that the ERP map topographies do not vary randomly in time, but remains quasi-stable over 20–100 ms functional microstates—that is, the ERP components, before rapidly switching to other stable periods (Lehmann and Skrandies 1980; Pascual-Marqui et al. 1995; Cacioppo et al. 2014). As in previous literature with the same analysis (e.g., Laganaro et al. 2012; Fargier and Laganaro 2016; Maitre et al. 2017), the optimal number of clusters that explained the best the grand-average data sets across conditions was identified using a modified version of the cross-validation criterion combining a cross-validation criterion and the Krzanovski-Lai criterion (Tibshirani and Walther 2005; see also Murray et al. 2008). This analysis enabled identifying the N2 and P3 ERP components’ onsets in our data in a data-driven manner for all conditions, further enabling the component-locking.

#### Global ERP Analyses

Modulations of the strength of the electric field at the scalp were analyzed using the GFP index (Lehmann and Skrandies 1980; Koenig and Melie-García 2010; Koenig et al. 2011). GFP is calculated as the spatial standard deviation of the electric field (i.e., the root-mean-square of the difference between two normalized vectors computed across the entire electrode set). Larger GFP amplitudes indicate stronger electric fields which can arise either from increase in the synchronization or in the extent of the neural sources underlying the scalp-recorded activity (Michel and Murray 2012).

Modulations of the topography of the electric field at the scalp were analyzed using the global map dissimilarity (GMD) index (Lehmann and Skrandies 1980). GMD indexes differences in the configuration between two electric fields and is calculated as the root-mean-square of the difference between the potentials measured at each electrode for the different experimental conditions normalized by instantaneous GFP. Because changes in topography forcibly follow from changes in the configuration of the underlying active sources (Lehmann and Skrandies 1980), topographic modulations reveal when distinct brain networks are activated across experimental conditions.

Since the GFP is insensitive to spatial (i.e., topographic) change in the potential distribution, and that GMD is calculated on GFP-normalized data, the GFP and GMD are orthogonal measures and can thus be interpreted separately.

GFP and GMD were compared across experimental conditions at each time frame using nonparametric randomization statistics (Monte-Carlo bootstrapping): the differences in GFP and GMD between the experimental conditions were compared with a distribution of the differences derived from permuting 5000 times the conditions’ label of the data for each participant (i.e., to which experimental condition they corresponded; Murray et al. 2008; Koenig et al. 2011; Tzovara et al. 2012). The probability of obtaining a GMD and delta GFP values from the permutations higher than the measured value was then determined. The threshold for statistical significance was set at *P* < 0.05, and to correct for multiple comparison and temporal autocorrelation we set a minimal duration threshold for a significant effect to be considered. This minimal duration threshold was determined as the shortest duration of consecutive significant time-points that can be expected under the null-hypothesis (shuffled data) with a probability of 0.05 (Nichols and Holmes 2002; Koenig and Melie-García 2010; Koenig et al. 2011).

The ERP analyses were used to identify the periods of interest (POI) defined by sustained significant age × session and training × session interactions. Analyses at the source level were then computed during these POIs.

#### Electrical Source Estimations

Brain sources of ERP modulations were estimated using a distributed linear inverse solution model (a minimum norm inverse solution) combined with the local autoregressive average (LAURA) regularization approach, which describes the spatial gradient across neighboring solution points (Menendez et al. 2001; Grave De Peralta Menendez et al. 2004). LAURA enables investigating multiple simultaneously active sources and selects the configuration of active brain networks that better mimics biophysical behavior of neural fields. LAURA uses a realistic head model, and the solution space included 3005 nodes, selected from a grid equally distributed within the gray matter of the Montreal Neurological Institute’s average brain. The head model and lead field matrix were generated with the spherical model with anatomical constraints (SMAC; Spinelli et al. 2000). As an output, LAURA provides current density measures; their scalar values were evaluated at each node. Assessments of the localization accuracy of this inverse solution by fundamental and clinical research indicate that the estimations and the results of their statistical analyses can be confidently interpreted at the resolution of the grid size (here 6 mm; e.g., Menendez et al. 2001; Michel et al. 2004; Gonzalez Andino, Michel, et al. 2005a; Gonzalez Andino, Murray, et al. 2005b). To correct for multiple testing and spatial autocorrelation, we applied a spatial-extent correction (Ke) of ≥15 contiguous nodes with a *P*-value < 0.05. This spatial criterion was determined using the AlphaSim program (available at http://afni.nimh.nih.gov) and assuming a spatial smoothing of 6 mm FWHM. This program applies a cluster randomization approach. The 10 000 Monte-Carlo permutations performed on our lead field matrix revealed a false positive probability of <0.005 for a cluster greater than 15 nodes.

The ERPs were averaged for the POIs determined by the ERP analyses, their sources calculated and then submitted to the same two mixed age × session and training × session ANOVAs as for the ERP analyses, again focusing on the interaction terms.

## Results

### Data Loss and Block Exclusion at Pre- and Post-training Sessions

An average of 0.2% of trials were excluded after removing hit and FA trials with RTs below 100 ms. One block was excluded from the EEG data because of corrupted data files. The counts of excluded blocks based on the outlier response thresholds are reported in Table 3.

**Table 3:** Number of excluded blocks

Excluded block count (%)	Young Go/NoGo training	Older Go/NoGo training	Older 2-back training
	17 (4.4%)	11 (3.2%)	7 (2.1%)

### Sanity Checks

#### Performance Improvement During Training

**Figure 2:**
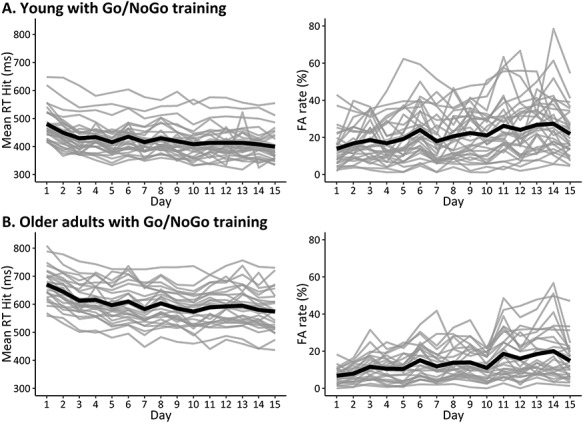
Change in behavioral performance over the 15 days of intervention for the two age groups (*A*. Young; *B*. Older). The group mean is in bold, and the individual data points in gray. One young participant did not complete his last day of training and one older participant did not complete his last 2 days of training.

Detailed results are reported in Table 4 and Figure 2. There was a decrease in RT in both groups, as indexed by significant negative linear regressions for both the young (b1 = −3.69, *t*(477) = −6.32, *P* < 0.001, *r* = −0.278) and older participants (b1 = −4.81, *t*(431) = −6.32, *P* < 0.001, *r* = −0.305).

**Table 4:** Linear regression of In-game performance

	Young Go/NoGo training (*n* = 32)	Older Go/NoGo training (*n* = 29)
RT hit (ms)	b0 = 453.4	b0 = 640.09
	b1 = −3.69	b1 = −4.81
	*P* < 0.001	*P* < 0.001
	*r* = −0.278	*r* = −0.305
FA rate (%)	b0 = 15.14	b0 = 7.58
	b1 = 0.75	b1 = 0.73
	*P* < 0.001	*P* < 0.001
	*r* = 0.243	*r* = 0.319

Notes: b0 = intercept, b1 = slope, *P* = *P*-value of the slope, *r* = linear correlation of the slope.

There was an increase in FA rate in both groups, as indexed by significant positive linear regressions in both the young (b1 = 0.75, *t*(477) = 5.48, *P* < 0.001, *r* = 0.243) and older participants (b1 = 0.73, *t*(431) = 6.98, *P* < 0.001, *r* = 0.319).

The results of the comparison between the young and older fit, and of the change in 2-back performance in the older 2-back training group are reported in the Supplementary Materials.

#### Sensitivity of the Executive Tasks to Aging

The Go/NoGo task was sensitive to the effect of aging: we replicated the typical slowing of response speed during executive tasks observed in older adult populations. For the RT, older adults were slower than young adults at the pretraining session (*t*(49) = −8.37, *P* < 0.001, *r* = 0.77). For the FA rate, we did not find any difference between the young and older participants (*t*(59) = 1.22, *P* = 0.23, *r* = 0.16).

#### Compliance to Instructions

We controlled that the participants followed the instruction to respond as fast as possible and that the task loaded as expected on the motor inhibition executive component with the following analyses: At the Go/NoGo task baseline, older and young adults had respectively an average of 439 and 389 ms RT on hit trials, and 12.3 and 14.8% of FA rate (Tables 6 and 8). Please see Supplementary Materials for further control analyses.

## Contrast 1: The Effect of the Go/NoGo Versus 2-back Training in Older Adults

### Session (Pre-; Post-training) by Training (Go/NoGo Training; Control 2-back Training) interaction

#### Questionnaires

The 2-back training resulted in larger improvement in QoL than the Go/NoGo training, although the effect size was small (*F*(1,54) = 5.62, *P* = 0.021, η_G_^2^ = 0.014; Table 5).

**Table 5:** Effect of the Go/NoGo versus 2-back training on the older group impulsivity (BIS-11) and QoL

	Older Go/NoGo training (*n* = 29)		Older 2-back training (*n* = 28)				
Mean ± SD pre- post-*t*-test	Pre-training	Post-training	Pre-training	Post-training	Training main effect	Session main effect	Training × Session interaction
BIS-11	2.01 ± 0.24	1.98 ± 0.28	2.02 ± 0.22	1.99 ± 0.23	*P* = 0.894	*P* = 0.351	*P* = 0.998
	*P* = 1		*P* = 1		η_G_^2^ = 0.000	η_G_^2^ = 0.004	η_G_^2^ = 0.000
	*r* = 0.06		*r* = 0.06				
QoL	7.92 ± 0.75	7.74 ± 0.69	7.56 ± 1.05^*^	7.76 ± 0.88	*P* = 0.471	*P* = 0.737	*P* = 0.021
	*P* = 0.73		*P* = 0.73		η_G_^2^ = 0.008	η_G_^2^ = 0.000	η_G_^2^ = 0.014
	*r* = 0.12		*r* = 0.11				

Note: ^*^One participant from the 2-back intervention did not complete the QoL questionnaire (*n* = 27 for this contrast).

### Behavior

**Figure 3:**
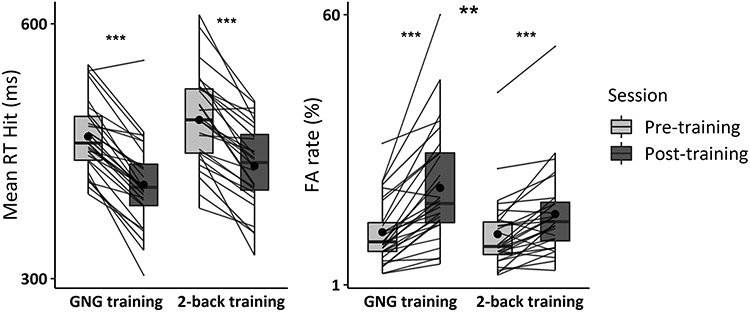
Behavioral performance during the pre- and post-training Go/NoGo task. The response time on hit trials and the false alarm rate of the older adults in the Go/NoGo and the 2-back training group are represented. Individual data points, means (bold circle), medians, first and third quartiles (horizontal bars), and the 1.5 interquartiles range (whiskers) are represented. ^*^P < 0.05, ^*^^*^P < 0.01, ^*^^*^^*^P < 0.001.

The full results of the session (pre; post-training) × training (Go/NoGo; control 2-back) design are reported in Table 6 and Figure 3. We describe in the results below only the interaction term of interest.

For the RT, there was no session by training interaction. BF analyses support this absence of interaction (BF_01_ = 3.12).

For the FA rate, there was a session by training interaction driven by a moderately larger increase in FA rate in the Go/NoGo than the 2-back training group (*F*(1,55) = 8.96, *P* = 0.004, η_G_^2^ = 0.02).

Given the pattern of decrease in RT and increase in FA rate revealed by the main effects of sessions, we investigated whether a speed accuracy trade-off took place by computing the correlation between the decrease in RT between the pre- and post-training session and the increase in FA rate (Supplementary Fig. 3). We found a significant negative linear correlation for both the older group with Go/NoGo training (*r*(27) = −0.45, *P* = 0.015) and with the 2-back training (*r*(26) = −0.45, *P* = 0.015). The more the RT decreased with training, the more the inhibition error rate increased.

### Electrical Neuroimaging

**Figure 4:**
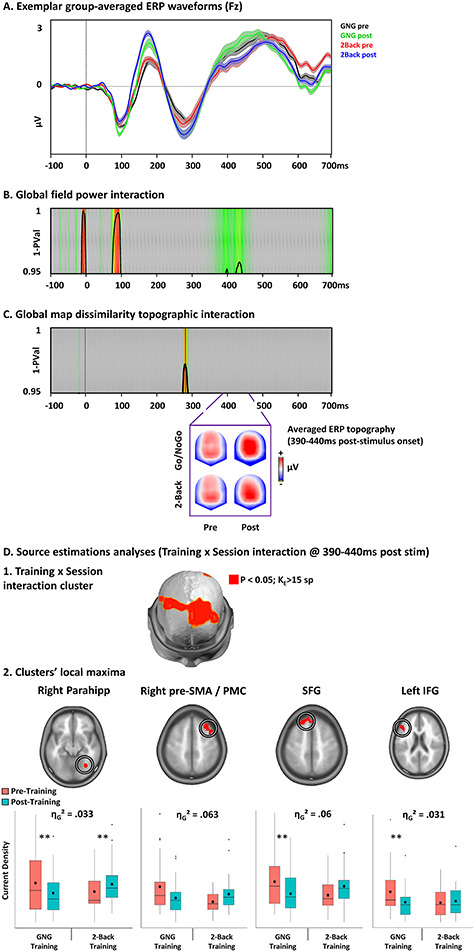
Electrical neuroimaging results: Training by Session interaction. (*A*) Exemplar group-average ERPs for correct NoGo trials in the older adults Go/NoGo and 2-back training groups for the pre- and post-training sessions. (*B* and *C*) Results of the GFP (*B*) and of the GMD topographic (*C*) training by session interaction revealed a sustained significant GFP but not topographic interaction during the P3 ERP component. The topographies of the ERP averaged over the period of GFP modulation are represented nasion upward for the four experimental conditions. (*D*) Source estimation analyses over the period of interest defined in the analyses in the sensor space. The plots represent the means (bold circle), medians, first and third quartiles (horizontal bars), and minimal–maximal values (whiskers) of the current densities at the clusters’ local maxima (i.e., the solution points with the lowest *P*-value) showing the training by session interaction. SMA: supplementary motor area; PMC: premotor cortex; IFG: inferior frontal gyrus; ^*^P < 0.05, ^*^^*^P < 0.01, ^*^^*^^*^P < 0.001.

Results are reported in Figure 4.

There was a GFP session by training type interaction during the P3 component (390–440 ms), without concomitant topographic modulation. Visual inspection of the ERP topography over this period indicates that this effect as mostly driven by a change in the GFP of the Go/NoGo training group.

Source estimations analyses localized this interaction in the right parahippocampal gyrus, right presupplementary motor area (pre-SMA), left superior frontal gyrus, and left IFG.

In the parahippocampal gyrus, the interaction was driven by a decrease in activity in the Go/NoGo training group and an increase in activity in the 2-back training group (η_G_^2^ = 0.033). In the right pre-SMA, the interaction was driven by a decrease in activity in the Go/NoGo training group and an increase in activity in the 2-back training group (η_G_^2^ = 0.063). In the left superior frontal gyrus, the interaction was driven by a decreased activity in the Go/NoGo training group and an increase in activity in the 2-back training group (η_G_^2^ = 0.06). In the left IFG, the interaction was driven by a decrease in activity in the Go/NoGo training group without change in the 2-back training group (η_G_^2^ = 0.031).

## Contrast 2: The Effect of the Go/NoGo Training in the Older Versus Young Adults

### Session (Pre-; Post-training) by Age (Young; Older Adults) interaction

#### Questionnaires

The Go/NoGo training had an opposite effect in the young and older groups. It was associated with an increase in the Barratt impulsivity measure in the Young group, but with a decrease in the older group, although the interaction effect size was small (*F*(1,59) = 5.04, *P* = 0.028, η_G_^2^ = 0.01; Table 7).

**Table 6:** Go/NoGo task behavioral performance (Training x Session)

	Older Go/NoGo training (*n* = 29)		Older 2-back training (*n* = 28)				
Mean ± SD pre- post-*t*-test	Pre-training	Post-training	Pre-training	Post-training	Training main effect	Session main effect	Training × session interaction
RT hit (ms)	467.3 ± 42.2	410.6 ± 50.8	486.9 ± 55	432.7 ± 49.6	*P* = 0.099	*P* < 0.001	*P* = 0.767
	*P* < 0.001		*P* < 0.001		η_G_^2^ = 0.044	η_G_^2^ = 0.245	η_G_^2^ = 0.000
	*r* = 0.89		*r* = 0.86				BF01 = 3.12
FA rate (%)	12.5 ± 7.2	22.2 ± 12.5	12.1 ± 8.1	16.5 ± 9.7	*P* = 0.204	*P* < 0.001	*P* = 0.004
	*P* < 0.001		*P* < 0.001		η_G_^2^ = 0.026	η_G_^2^ = 0.122	η_G_^2^ = 0.02
	*r* = 0.77		*r* = 0.69				

**Table 7:** Effect of the training on the older versus the young group impulsivity and QoL

	Young Go/NoGo training (*n* = 32)		Older Go/NoGo training (*n* = 29)				
Mean ± SD pre- post-*t*-test	Pre-training	Post-training	Pre-training	Post-training	Age main effect	Session main effect	Age × Session interaction
BIS-11	2.04 ± 0.29	2.12 ± 0.29	2.01 ± 0.24	1.98 ± 0.28	*P* = 0.22	*P* = 0.285	*P* = 0.028
	*P* = 0.54		*P* = 0.68		η_G_^2^ = 0.022	η_G_^2^ = 0.002	η_G_^2^ = 0.01
	*r* = 0.06		*r* = 0.06				
QoL	7.26 ± 0.9	6.98 ± 0.93	7.92 ± 0.75	7.74 ± 0.69	*P* = 0.001	*P* = 0.006	*P* = 0.498
	*P* = 0.45		*P* = 0.45		η_G_^2^ = 0.161	η_G_^2^ = 0.019	η_G_^2^ = 0.001
	*r* = 0.15		*r* = 0.12				

### Behavior

**Figure 5:**
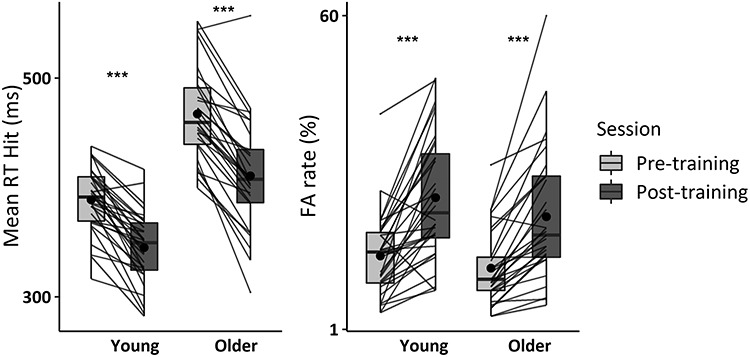
Behavioral performance during the pre- and post-training Go/NoGo task. The response time on hit trials and the false alarm rate of the young and older adults with Go/NoGo training are represented. Individual data points, means (bold circle), medians, first and third quartiles (horizontal bars), and the 1.5 interquartiles range (whiskers) are represented. ^*^P < 0.05, ^*^^*^P < 0.01, ^*^^*^^*^P < 0.001.

The results are reported in Table 8 and Figure 5.

For the RT, there was no session by age interaction, with the BF analysis indicating a 1.25 likelihood of the null as compared with the alternative hypothesis (BF_01_ = 1.25).

For the FA rate, there was no session by age interaction, with evidence for an absence of interaction at the BF analysis (BF_01_ = 3.32).

Given the pattern of decrease in RT and increase in FA rate revealed by the main effects of sessions, we investigated whether a speed accuracy trade-off took place by correlating the decrease in RT between the pre- and post-training session to the increase in FA rate. We found a significant negative correlation for both the young (*r*(30) = −0.69, *P* < 0.001) and older group with Go/NoGo training (*r*(27) = −0.45, *P* = 0.015). The more the RT decreased post-training, the more error participants committed in both groups.

### Electrical Neuroimaging

**Figure 6:**
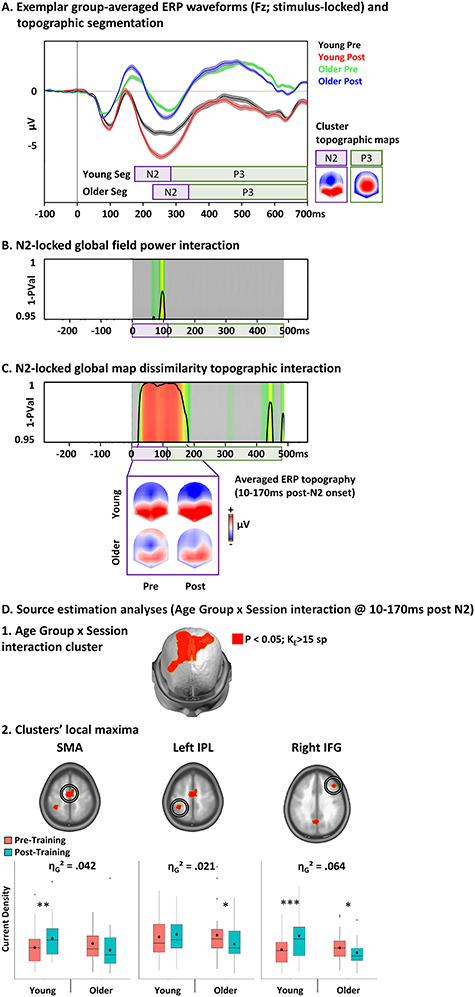
Electrical neuroimaging results: Age by Session interaction. *A*. Exemplar group-average stimulus-locked ERPs for correct NoGo trials in the two age groups with Go/NoGo training for the pre- and post-training sessions. The template topographic maps of the temporal segmentation analysis used to define the periods of the N2 and P3 components are represented nasion upward. *B* and *C*. Results of the GFP (*B*) and of the GMD topographic (*C*) Age by session interaction revealed a sustained significant topographic but not GFP interaction during the N2 ERP component. The topographies of the ERP averaged over the period of the topographic modulation are represented nasion upward for the four experimental conditions. (*D*) Source estimation analyses over the period of interest defined in the analyses in the sensor space. The plots represent the means (bold circle), medians, first and third quartiles (horizontal bars), and minimal–maximal values (whiskers) of the current densities at the clusters’ local maxima (i.e., the solution points with the lowest *P*-value) showing the age by session interaction. SMA: supplementary motor area; IPL: inferior posterior lobe; IFG: inferior frontal gyrus; ^*^P < 0.05, ^*^^*^P < 0.01, ^*^^*^^*^P < 0.001.

Results are reported in Figure 6.

**Table 8:** Go/NoGo task behavioral performance (Age by Session)

	Young Go/NoGo training (*n* = 32)		Older Go/NoGo training (*n* = 29)				
Mean ± SD pre- post-*t*-test	Pre-training	Post-training	Pre-training	Post-training	Age main effect	Session main effect	Age × session interaction
RT hit (ms)	388.9 ± 29	345.1 ± 32.4	467.3 ± 42.2	410.6 ± 50.8	*P* < 0.001	*P* < 0.001	*P* = 0.09
	*P* < 0.001		*P* < 0.001		η_G_^2^ = 0.47	η_G_^2^ = 0.3	η_G_^2^ = 0.01
	*r* = 0.84		*r* = 0.89				BF01 = 1.25
FA rate (%)	14.8 ± 7.7	25.8 ± 11.7	12.5 ± 7.2	22.1 ± 12.5	*P* = 0.205	*P* < 0.001	*P* = 0.593
	*P* < 0.001		*P* < 0.001		η_G_^2^ = 0.022	η_G_^2^ = 0.214	η_G_^2^ = 0.001
	*r* = 0.75	*r* = 0.77				BF01 = 3.32

After locking the ERPs on N2’s onset (see Supplementary Fig. 1 for groups’ onset), we observed a sustained topographic session by age group interaction during the N2 and early P3 components (10–170 ms post-N2). Visual inspection of the ERP topography over this period indicated that the interaction was mostly driven by a change in topography in both the young and older population.

Source estimation localized this interaction in the bilateral SMA, left inferior parietal lobe (IPL), and rIFG. In the SMA, the interaction was driven by an increased activity in the young group and a decreased activity in the older group (η_G_^2^ = 0.042). In the left IPL, the interaction was driven by a decreased activity in the older group without change in the young group (η_G_^2^ = 0.021). In the rIFG, the interaction was driven by an increased activity in the young group and a decreased activity in the older group (η_G_^2^ = 0.064).

## Discussion

We identified the behavioral and brain functional plastic modifications induced by a 3-week home-based gamified ICT (ICT vs. a 2-back working memory training) in a group of older adults. As a second step, we revealed how aging interacted with the ICT by comparing its effects with those observed in a group of young adults participating in the same inhibition training intervention.

We found that older populations show a preserved capacity for functional modification and behavioral change with ICT. However, while as compared with a 2-back working-memory training the ICT in the older adult induced purely quantitative reductions of prefrontal activity during the P3 inhibition-related ERP component, as compared with young adults the ICT induced distinct configurational modifications in older adults medial–frontal N2 conflict monitoring activity.

### Inhibitory Control Training Alters Performance and Modifies Response Strategies in Older as in Young Populations

As expected, and replicating previous literature, we observed a globally lower performance in the older than young adult group in the IC tasks used for the training and the assessment of its effect (Nielson et al. 2002; Vallesi et al. 2011; Heilbronner and Münte 2013; Hong et al. 2014). These results confirm that our intervention adequately targeted the inhibition processes typically declining with aging.

The lower performance in the older group was characterized by slower RT with similar FA rate both at the pre- and post-training sessions. Interestingly, the training resulted in a corresponding decrease in response time with an increase in false alarm rates in the young and older adults training groups (nonsignificant interaction factor for both RT and FA rate), meaning that despite a difference in baseline performance, they had a similar performance improvement in both quantitative and qualitative terms. We interpret this as an evidence that, despite the age-related cognitive deterioration, a relevant potential for functional improvement is still preserved in older people, which is also in line with our findings of a preserved electrophysiological plasticity in this population. These changes in speed and accuracy correlated with each other, indicative of a trade-off toward more impulsive response strategies. Although the autoadaptive response-time threshold may have contributed to this shift by maintaining a constant pressure on response speed, it does not account for the whole pattern of performance change because accuracy was also emphasized via performance-based feedbacks. This assumption is further supported by our finding for smaller effect sizes of the increase in FA rate than those of the decrease in RT, suggesting that the training still improved performance. Indeed, according to the race model, a larger increase in response speed than in accuracy necessarily indicates that the speed of inhibition also increases (Logan et al. 2014; Verbruggen and Logan 2015; see Manuel et al. 2010; Benikos et al. 2013; Chavan et al. 2015; Hartmann et al. 2016 for similar behavioral patterns with Go/NoGo training). Importantly, changes in response strategy and performance improvement are not mutually exclusive, and likely both took place in the present study. While changes in response strategy with ICT were not hypothesized in the present study, they might be considered as potential hypotheses in future investigation and even targeted and promoted by emphasizing response speed or accuracy depending on the specific aim of the intervention.

Regarding more specifically the comparison between the 2-back versus ICT older adult group, we found that while the reductions in response time were comparable between the two groups, the ICT group showed a larger increase in false alarms rates. We interpret this pattern as a larger shift to impulsive response mode in the ICT group because they showed both more FA and shorter RT in the post-training session.

When comparing changes in inhibition performance between the two age groups, we found that the ICT had equivalent behavioral effect despite initially slower response speed in the older adult group, with this pattern manifesting both in the gamified Go/NoGo training task and the Go/NoGo task given at pre- and post-training. These findings suggest that the capacity to improve performance with ICT is possibly modified but certainly not reduced by the prefrontal structural deterioration or the compensatory functional reorganization associated with healthy aging.

The questionnaires on impulsivity and QoL finally revealed that the ICT did not influence IC capacities beyond the trained tasks. Indeed, while we observed a session by age interaction for the impulsivity trait and a session by training interaction for the QoL, the effect sizes were so small that the interactions cannot, in our views, be considered as meaningful. This is in line with current evidence for highly specific effect of executive training (Sala and Gobet 2019). We cannot exclude, however, that a lack of sensitivity of our measure accounted for our null results. Future investigation may focus on well validated measure of real-life influence of low-level motor IC processes, though such metrics seem difficult to establish (Eisenberg et al. 2019).

### In Older Adults, Inhibitory Control Training Improves Central and Lateral Prefrontal Inhibition Processes

When comparing in older adults the effects of the ICT to those of a control 2-back working memory training, we found that it modulated the GFP of the P3 ERP component without influencing its topography. This pattern thus indicates that ICT influences response gain of the involved IC network, but not its configuration. Mechanistically, ICT in older adults thus results in a purely quantitative change in the activity of the inhibition networks. Such reduction in activity with training have been repeatedly observed with ICT and are thought to result from a neural sharpening process improving efficiency (e.g., Manuel et al. 2013; Chavan et al. 2015; Hartmann et al. 2016; Simonet et al. 2019 for corresponding effects).

The latency of the effect at 400 ms during the P3 component first indicates that the ICT modulated the implementation of the motor suppression command (Smith et al. 2008; Albert et al. 2013; Gajewski and Falkenstein 2013). Consistent with this interpretation and with previous localizations of the P3 generators, the statistical analyses of the source estimations over this period revealed a decrease in activity within the right preSMA/PMC, left superior and inferior frontal gyri, and right parahippocampal gyri modulation in the older adults with ICT.

These areas have been involved in IC in young adults (preSMA; Rubia et al. 2001; Mostofsky et al. 2003; Li et al. 2006; Simmonds et al. 2008; Xue et al. 2008; Swick et al. 2011) and/or have been shown to increase in activity with aging (Park and Reuter-Lorenz 2009); they are thus thought to reflect a compensation for the age-related deficits via “neurocognitive scaffolding” mechanisms (Park and Reuter-Lorenz 2009; see also Turner and Spreng 2012 for data on larger preSMA activity in older adults during conflict processing and IC).

We thus interpret our findings as revealing both an improvement in the functioning of the areas primarily involved in IC, and a reduction in compensatory activity with training. Given the proposed link between these functional changes with performance improvements and shifts in response strategy, they putatively support a better coping with task demands (e.g., Anguera et al. 2013; Heinzel et al. 2014).

### As Compared with Young Adults, Older Adults’ Medial Prefrontal Conflict Monitoring Shows a Different Susceptibility to Inhibitory Control Training

The difference in the effect of the ICT between the young and the older adults manifested as a topographic ERP interaction during the N2 and early P3 component, indicating that the training-induced distinct changes in the configuration of the inhibition networks between the young and older populations. Hence, aging does not simply influence the amplitude of the training-induced plastic changes, but also the underlying mechanism. This finding is compatible with previous evidence for an influence of baseline functional organization on executive control plasticity (Cabeza et al. 2002; Cappell et al. 2010; Hsieh and Fang 2012; Sebastian et al. 2013; Reuter-Lorenz and Park 2014; Coxon et al. 2016). Given the age-related structural deterioration of prefrontal cortices and the engagement of compensatory functional activity in the older population, functional remodeling induced by the ICT did not take place in the same way nor on the same networks in young and older adults. We advance that these factors account for the observed difference in the network reconfiguration. However, the neurophysiological mechanisms driving by these different functional changes remain to be determined by studies combining microstructural and functional measures.

Our findings for plastic changes during the N2 and early P3 indicate that aging modifies the sensitivity to ICT of the conflict monitoring and initiation of the inhibition command (Nieuwenhuis et al. 2003; Donkers and Van Boxtel 2004; Schmajuk et al. 2006; Enriquez-Geppert et al. 2010; Gajewski and Falkenstein 2013). Importantly, the N2 also indexes preparatory processes occurring before the actual control of the motor response, such as bottom-up attention of the detection of response conflict (Albert et al. 2013). As mentioned above, the period of the early P3 entails the implementation of the motor inhibition process (Smith et al. 2008; Albert et al. 2013; Gajewski and Falkenstein 2013). Interestingly, this result echoes findings from a previous paper of our group in which we observed, after a short 40 min ICT, a decreased activity in the SMA and an increased activity in the left VLPFC in the older adults compared with young adults during the N2 and P3 ERP components, respectively (Hartmann et al. 2019).

Furthermore, the location of the interactions in the SMA, rIFG, and left IPL is in line with previous literature showing localization of the sources of the N2 and P3 components in medial and ventrolateral prefrontal cortices (Hartmann et al. 2019). As also mentioned, activity in the preSMA and SMA has been associated with the preparation and the implementation of the motor suppression (Rubia et al. 2001; Mostofsky et al. 2003; Floden and Stuss 2006; Li et al. 2006; Simmonds et al. 2008; Xue et al. 2008; Chen et al. 2009; Swick et al. 2011), and correlates with inhibition performance (Li et al. 2006). Given the medial position of this cluster, our source localization algorithm may not have differentiated whether it was more lateralized toward one of the hemispheres. Given the typical control of motor activity by contralateral motor area, we suppose the left pre-SMA activity may have been predominantly modified by the training (Li et al. 2006). The rIFG constitutes the key node of motor response inhibition (Bernal and Altman 2009; Aron et al. 2014), and its microstructure is influenced by ICT in older adults (Kühn et al. 2017). As for the IPL, it is involved in sensorimotor integration, and contributes to the conscious perception of motor intention (Fridman et al. 2011; Desmurget and Sirigu 2012). Accordingly, the left lateralization of this effect likely follow from the involvement of the contralateral right hand during the task; the training may have modified differently stored movement representations and/or how they are used, which is compatible with our hypothesis for an differential effect of the training on processing phases related to how younger and older participants cope with task demands.

Our observation for increased activity in the young and decreased in the older adults suggests that training may have developed different coping strategies in these two groups; we speculate that in the older adults group, the training reduced the compensatory strategies engaged to better cope with the task’s demands (Turner and Spreng 2012 for a meta-analysis), whereas the increase in activity in the young adults might reflect that they recruited additional resources to cope with the increase in task difficulty during the training.

## Conclusion and Future Directions

Our collective findings finally indicate that while the ICT induces equivalent behavioral changes in older and young adults, the modulation of the IC neural processes associated with these alterations in performance differs qualitatively between the two groups. Hence, while the potential for functional improvement is preserved in older adults, predictions on the functional effect of executive control training in this population could not be readily derived from models developed in young populations. Our results also underline the state-dependency of training-induced plasticity in executive control, which strongly depends on baseline brain state.

In this regard, the training difficulty levels should be carefully chosen in ICT programs. Likewise, systematic investigation on the effect of adaptive algorithm for response time threshold, and of the type of feedback provided on accuracy, would be useful to optimize cognitive control intervention in older adults. Whether a given training intervention improve performance and/or influence response strategy indeed possibly depends on difficulty levels and on the aspect of the task emphasized by the instruction and in-task feedbacks.

To conclude, we would note that the home-based gamified intervention was well received in both young and older populations. Three weeks of training were achieved with almost no dropouts, supporting previous claims that gamification contributes to compliance to computerized cognitive interventions (Mishra et al. 2016). Moreover, the intervention successfully induced functional neuroplastic and behavioral improvements. This also demonstrate possible applicability of this method in cognitive rehabilitative settings, where it would represent a valuable approach to provide an effective, easy to implement, well accepted, home-based treatment for various target populations.

## Supplementary Material

Supplementary material can be found at *Cerebral Cortex* online.

## Notes

We thank Amanda Capobianco for her help in data collection. The intervention application The Diner has been developed in collaboration with Maurizio Rigamonti for the programming and Pauline Rossel for the artistic direction. The Cartool software (brainmapping.unige.ch/cartool) has been programmed by Denis Brunet, from the Functional Brain Mapping Laboratory, Geneva, Switzerland, and is supported by the Center for Biomedical Imaging (CIBM) of Geneva and Lausanne. The STEN toolbox (http://doi.org/10.5281/zenodo.1164038) has been programmed by Jean-Francois Knebel and Michael Notter, from the Laboratory for Investigative Neurophysiology (the LINE), Lausanne, Switzerland, and is supported by the Center for Biomedical Imaging (CIBM) of Geneva and Lausanne and by National Center of Competence in Research project “SYNAPSY—The Synaptic Bases of Mental Disease”; project no. 51AU40_125759.* Conflict of Interest:* None declared.

## Funding

Velux Stiftung (grant #1078 to L.S.); the Swiss National Science Foundation (grant #320030_175469 to L.S.); the Research Pool of the University of Fribourg (to L.S.).

## Supplementary Material

Supplementary_Materials_bhaa259
