# Supplementary material for: Aging Modulates Prefrontal Plasticity Induced by Executive Control Training
Source: Cereb Cortex. 2020 Sep 15;31(2):809–25. doi: 10.1093/cercor/bhaa259 (PMC7786350; doi:10.1093/cercor/bhaa259)
Supplement: Supplementary_Materials_bhaa259 [file supplementary_materials_bhaa259.docx]

# Supplementary material

**Temporal topographic segmentation to lock the ERP on the N2 onset**

To identify the periods of the N2 ERP components, we submitted the group-averaged ERP data of the young and the older adults to hierarchical clustering based on an atomize and agglomerate analysis (Brunet et al., 2011; Murray et al., 2008). This approach is based on evidence that the ERP map topography does not vary randomly across time, but remains quasi-stable over 20–150 ms functional microstates – i.e. the ERP components- before rapidly switching to other stable periods (Cacioppo, Weiss, Runesha, & Cacioppo, 2014; Lehmann & Skrandies, 1980). As in previous literature with the same analysis (e.g. Laganaro, Valente, & Perret, 2012; Maitre et al., 2017), the optimal number of clusters that explained the best the grand-average data sets across conditions was identified using a modified version of the cross-validation criterion combining a cross-validation criterion and the Krzanovski-Lai criterion (see Murray et al., 2008). The computation resulted in a maximum fit of five clusters for each grand averaged ERP (supp. figure 1).

*Supplementary figure 1. Schematic representation of the cluster-based segmentation’s results for both age groups at both sessions. N2’s onsets are: Young Pre-Training: 171ms; Young Post-Training: 176ms; Older Pre-Training: 234ms; Older Post-Training: 229ms.*


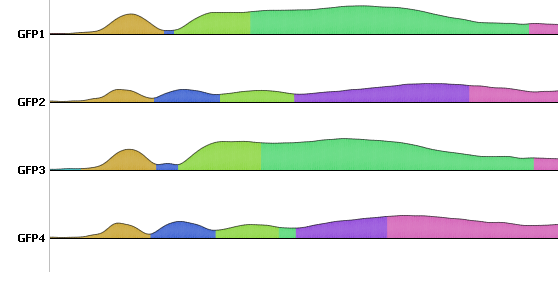


Young Pre-Training
N2’s onset: 171 ms

Older Pre-Training
N2’s onset: 234 ms

Older Post

Young Post-Training
N2’s onset: 176 ms

Older Post-Training
N2’s onset: 229 ms

**N2**

**N2**

**N2**

**N2**

**P3**

**P3**

**P3**

**P3**

**700ms**

**0**

**100**

**200**

**300**

**400**

**500**

**600**

Before the N2 component, we can observe in this segmentation a cluster greatly varying in length with age, shifting the N2 and P3 components’ onsets. As this cluster is assumed to be a component related to the stimulus perceptual discrimination, and as we are focused in comparing the inhibition-related components, we decided to lock the ERPs at N2’s onset based on this segmentation.

**References**

Cacioppo, S., Weiss, R. M., Runesha, H. B., & Cacioppo, J. T. (2014). Dynamic spatiotemporal brain analyses using high performance electrical neuroimaging: Theoretical framework and validation. Journal of Neuroscience Methods, 238, 11–34. https://doi. org/10.1016/j.jneumeth.2014.09.009

Laganaro, M., Valente, A., & Perret, C. (2012). Time course of word production in fast and slow speakers: A high density ERP topographic study. Neuroimage, 59, 3881–3888. <https://doi.org/10.1016/j.neuroimage.2011.10.082>.

Lehmann, D. (1987). Principles of spatial analysis. In A. S. Gevins, & A. Remond (Eds.), Handbook of electroencephalography and clinical neurophysiology. Methods ofanalysis of brain electrical and magnetic signals (pp. 309–354).

Lehmann, D., & Skrandies, W. (1980). Reference-free identification of components of checkerboard-evoked multichannel potential fields. Electroencephalography and Clinical Neurophysiology, 48, 609–621. https://doi.org/10.1016/0013-4694(80) 90419-8.

Maitre, N. L., Key, A. P., Chorna, O. D., Slaughter, J. C., Matusz, P. J., Wallace, M. T., & Murray, M. M. (2017). The dual nature of early-life experience on somatosensory processing in the human infant brain. Current Biology, 27, 1048–1054. https://doi. org/10.1016/j.cub.2017.02.036.

Murray, M. M., Brunet, D., & Michel, C. M. (2008). Topographic ERP analyses: A step-bystep tutorial review. Brain Topography, 20, 249–264. https://doi.org/10.1007/ s10548-008-0054-5.

**Generalized Linear Model to compare the evolution of performance during training**

Potential differences between the Young and Older training in-game performance models (see Sanity Checks section of the main manuscript) were assessed using the interaction term of a Day by Age (Young vs. Older) generalized linear model (GLM).

## There was no evidence for a difference in the effect of training between the two groups (no Day by Age interaction in the GLM; supp. table 1) for both the RT Hit and FA rate.

Supplementary table 1. Generalized linear model interaction of in-game performance

|  | **Day x Age GLM interaction** |
| --- | --- |
| **RT Hit (ms)** | b1 = 1.11  p = .225 |
| **FA rate (%)** | b1 = 0.02  p = .91 |

**Performance of Older adults with Control 2-back training**

Detailed results are reported in the supplementary table and figure 2. For the Older adults with control 2-back training, there was an improvement in performance in all behavioral measures, as indexed by a decrease in response times (RT; r(27) = -.44, p < .001), a decrease in false alarm (FA) rates (r(27) = -.39, p < .001), and an increase in the global performance index (Hit – FA rate; r(27) = .56, p < .001).

Supplementary table 2. Linear regression of the Older adults with Control 2-back training’s in-game performance

|  | **Older adults with 2-back training (n = 28)** |
| --- | --- |
| **RT Hit (ms)** | b0 = 719.82  b1 = -5.86  p < .001  r = -.44 |
| **FA rate (%)** | b0 = 18.92  b1 = -0.75  p < .001  r = -.38 |
| **Hit - FA rate (%)** | b0 = 52.02  b1 = 2.32  p < .001  r = .56 |

b0 = intercept, b1= slope, p = p-value of the slope, r = linear correlation of the slope).

#
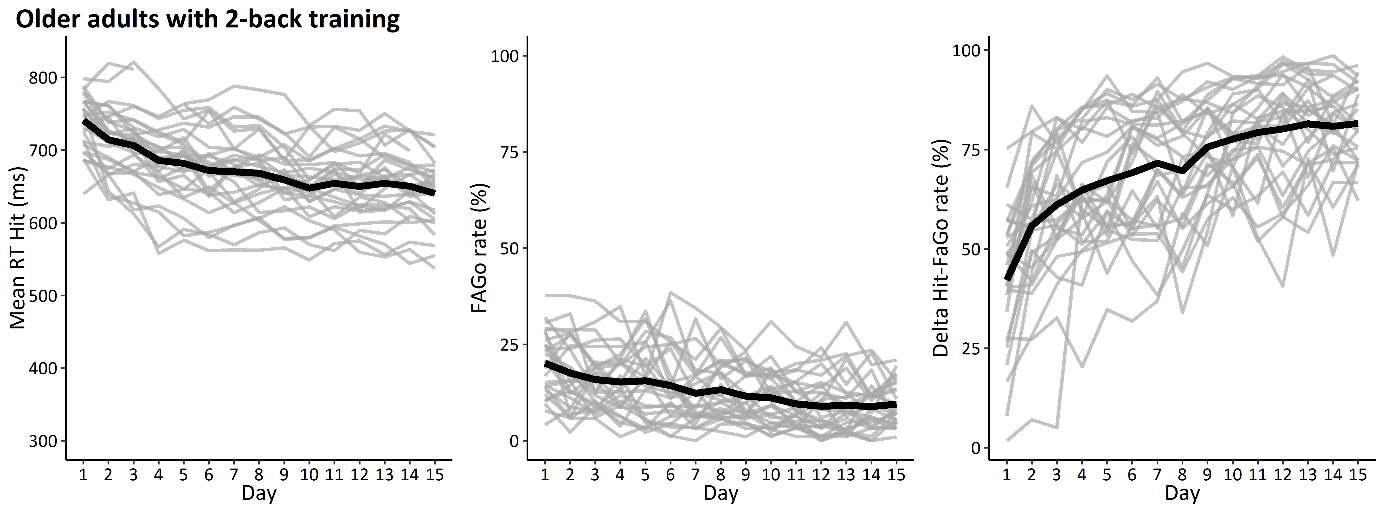


Supplementary figure 2. Schematic representation of the evolutions in behavioral performances over the 15 days of intervention for the Older adults with Control 2-back training. The group’s mean is bold, and the individual data points are grey.

Speed accuracy trade-off

Speed accuracy trade-offs could be observed in all age and training groups during the pre- and post-training Go/NoGo task. These trade-offs are indexed by negative correlations between their relative decrease in RT increase in FA rate for the young adults group (r(30) = -.69, p < .001), the older adults with Go/NoGo training group (r(27) = -.45, p = .015), and the older adults with 2-back training group (r(26) = -.45, p = 0.15).


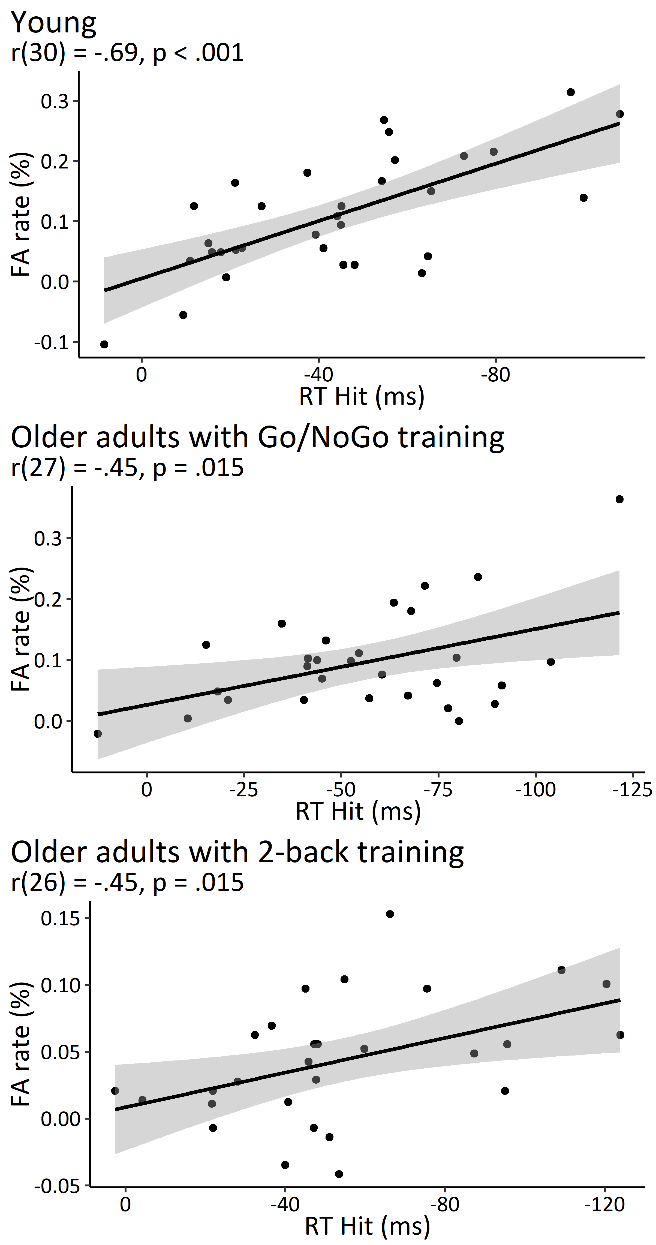


Supplementary Figure 3. Schematic representation of the correlations between the decrease in RT between the pre- and post- training session and the increase in FA rate. Individual data points (dots), and linear models with their standard errors (grey area) are represented.

Pre-Post- Training performance at the 2-back task

Task procedure

After the Go/NoGo task, participants completed four blocks of a 2-back task. In this task, a button had to be pressed during a target item, if another stimulus of the target category was presented two items back (supp. figure 4). The stimuli and their categories, the number of trials per block, and the general task timeline were identical to the Go/NoGo task at the exception of the response time threshold that was much larger to avoid inducing motoric inhibition, i.e. 1.5*Median of the last five RTs.

Fixation Cross

1000-2000 ms

NoGo trial

Feedback

Response Time Limit

Inter-Stimulus Interval


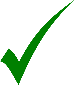


500 ms

250-1500 ms

350 ms

1000-2000 ms


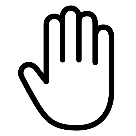


NoGo trial


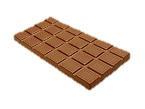


500 ms

*time*


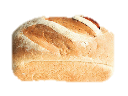

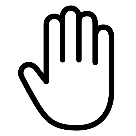


*Supplementary Figure 4. Timeline of a 2-back task trial.*

Behavioral dependent variables

The same variables as the Go/NoGo task were assessed. However, false alarms and correct rejections in the 2-back task were further split between responses to the NoGo stimuli that were in the target category (i.e. that might be responded to depending on the preceding trial sequence: FAGo and CRGo) and NoGo stimuli that were not in the target category (i.e. that never had to be responded to: FANoGo and CRNoGo).

The performance at this task was assessed with the mean RT of Hit trials, the mean FAGo rate, and the difference between the Hit rate (i.e., the opposite of Miss rate), and FAGo rate as a global measure of performance.

Behavioral data pre-processing

The data-processing was as described in the main manuscript, except for the criteria to exclude blocks, which were: FAGo rate above 0.7; Miss rate above 0.7; or FAGo, FANoGo and/or Miss rate above their respective intra-session median + 0.2.

Results

**Baseline Sanity Checks**

We replicated previous literature for slower response speed during executive tasks in older adults. For the RT, older adults were slower than young adults at the pre-training session (t(39) = -4.82, p < .001, r = .61). For the FAGo rate, we did not find any difference between the Young and Older participants (t(48) = -1.1, p = .28, r = .15). For the Hit - FAGo rate, older adults showed lower performance than the young adults (t(41) = -2.7, p = .009, r = .39). RTs were 100ms longer at the 2-back task compared to the Go/NoGo task for both Older and Young group, indicating less pressure to respond and thus a lower loading on inhibition, if any.

## Contrast 1: **The effect of the Go/NoGo vs 2-Back training in older adults**

The full results of the Session (Pre; Post-training) x Training (Go/NoGo; Control 2-back) design are reported supplementary table 3 and figure 5. We describe in the results below only the interaction term of interest.

For the RT, there was a Training by Session interaction driven by a larger decrease in RT in the 2-back than in the Go/NoGo training group (F(1,52) = 4.19, p = .046, η_G_² = .017).

For the FAGo rate, there was a Training by Session interaction driven by a larger decrease in error rate in the 2-back than in the Go/NoGo training group (F(1,52) = 6, p = .018, η_G_² = .025).

For the difference between Hit and FAGo rate, there was a Training by Session interaction driven by a larger increase in performance in the 2-back than in the Go/NoGo training group (F(1,52) = 5.19, p = .027, η_G_² = .017).

Supplementary table 3. 2-back task behavioral performance

|  | **Older Go/NoGo Training (n=27)** | | **Older 2-back Training (n=27)** | |  |  |  |
| --- | --- | --- | --- | --- | --- | --- | --- |
| **Mean ±SD pre- post- t-test** | **Pre-training** | **Post-training** | **Pre-training** | **Post-training** | **Training**  **Main Effect** | **Session**  **Main Effect** | **Training x Session**  **Interaction** |
| **RT Hit (ms)** | 542 ±97.2 | 459 ±72.6 | 574.8 ±109.9 | 447.6 ±51.4 | p = .606  η_G_² = .004 | p < .001  η_G_² = .28 | p = .046  η_G_² = .017 |
|  | p < .001  r = .77 | | p < .001  r = .83 | |  |  |  |
| **FAGo rate (%)** | 16.1 ±14.1 | 13 ±11.2 | 15.6 ±6.3 | 6.3 ±4.7 | p = .137  η_G_² = .033 | p < .001  η_G_² = .094 | p = .018  η_G_² = .025 |
|  | p = .15  r = .28 | | p < .001  r = .81 | |  |  |  |
| **Hit-FaGo rate (%)** | 65.8 ±20 | 75.5 ±14.2 | 68.3 ±13.1 | 85.4 ±6.5 | p = .084  η_G_² = .047 | p < .001  η_G_² = .185 | p = .027  η_G_² = .017 |
|  | p < .001  r = .64 | | p < .001  r = .83 | |  |  |  |


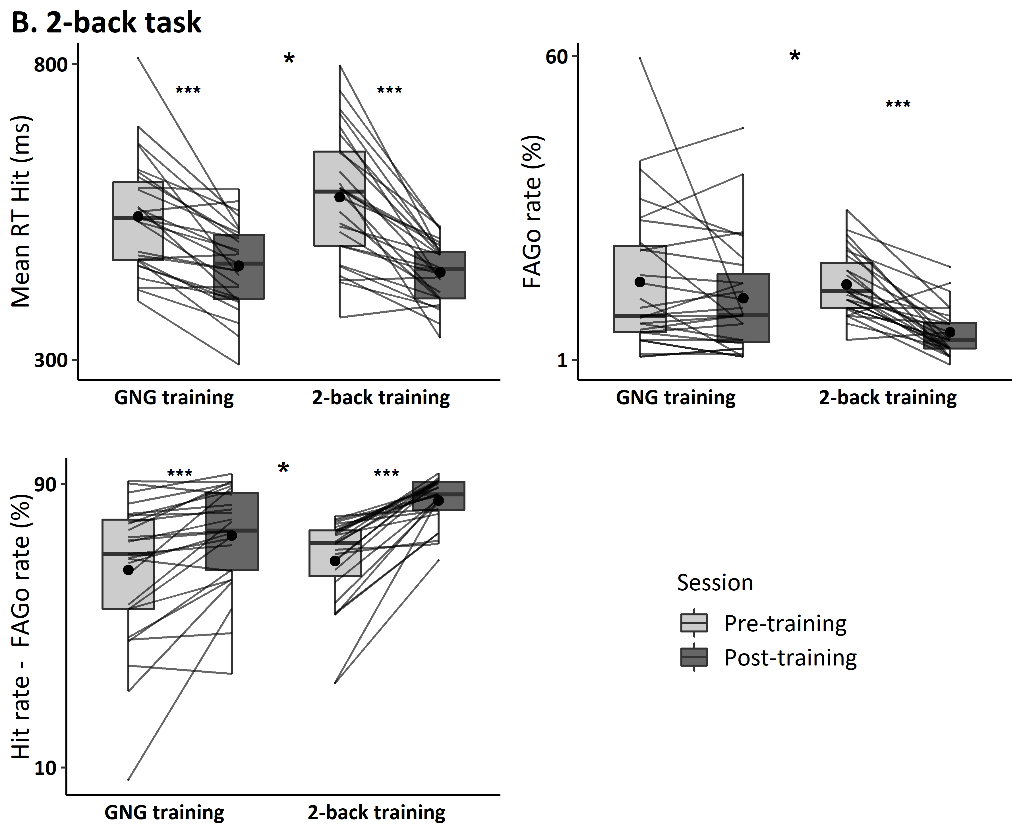


Supplementary figure 5. Behavioral performance during the 2-back task. Older adults with Go/NoGo and 2-back training are represented for all behavioral measures: Mean Response Time on Hit trials and False Alarm rate. Individual data points, means (bold circle), medians, first and third quartiles (horizontal bars), and the 1.5 inter-quartiles range (whiskers) are represented. *: p<.05, **: p<.01, ***: p<.001.

## Contrast 2: The effect of the Go/NoGo training in the Young vs. Older adults

The full results of the Session (Pre; Post-training) x Age (Young; Older adults) design are reported supplementary table 3 and figure 5. We describe in the results below only the interaction term of interest.

For the RT, there was an Age by Session interaction effect driven by a larger decrease in RT in the Older than Young group (F(1,57) = 11.6, p = .001, η_G_² = .035).

For the FAGo rate, there was no Session x Age interaction.

For the difference between Hit and FAGo rate, there was an Age by Session interaction effect driven by larger increase in performance for the Older than Young group (F(1,57) = 9.79, p = .003, η_G_² = .017).

Supplementary table 4. 2-back task behavioral performance

|  | **Young**  **(n=32)** | | **Older Go/NoGo Training (n=27)** | |  |  |  |
| --- | --- | --- | --- | --- | --- | --- | --- |
| **Mean ±SD pre- post- t-test** | **Pre** | **Post** | **Pre** | **Post** | **Age**  **Main Effect** | **Session**  **Main Effect** | **Age x Session**  **Interaction** |
| **RT Hit (ms)** | 440.9 ±53.6 | 408.4 ±38.9 | 542 ±97.2 | 459 ±72.6 | p < .001  η_G_² = .245 | p < .001  η_G_² = .158 | p = .001  η_G_² = .035 |
|  | p < .001  r = .61 | | p < .001  r = .77 | |  |  |  |
| **FAGo rate (%)** | 12.5  ±10.8 | 11.8 ±11 | 16.1 ±14.2 | 13  ±11.2 | p = .401  η_G_² = .011 | p = .098  η_G_² = .007 | p = .307  η_G_² = .003 |
|  | p = .51  r = .12 | | p = .31  r = .28 | |  |  |  |
| **Hit-FaGo rate (%)** | 77.7 ±12 | 79.5 ±14.5 | 65.8 ±20 | 75.5 ±75.5 | p = .039  η_G_² = .066 | p < .001  η_G_² = .035 | p = .003  η_G_² = .017 |
|  | p = .2  r = .23 | | p < .001  r = .64 | |  |  |  |


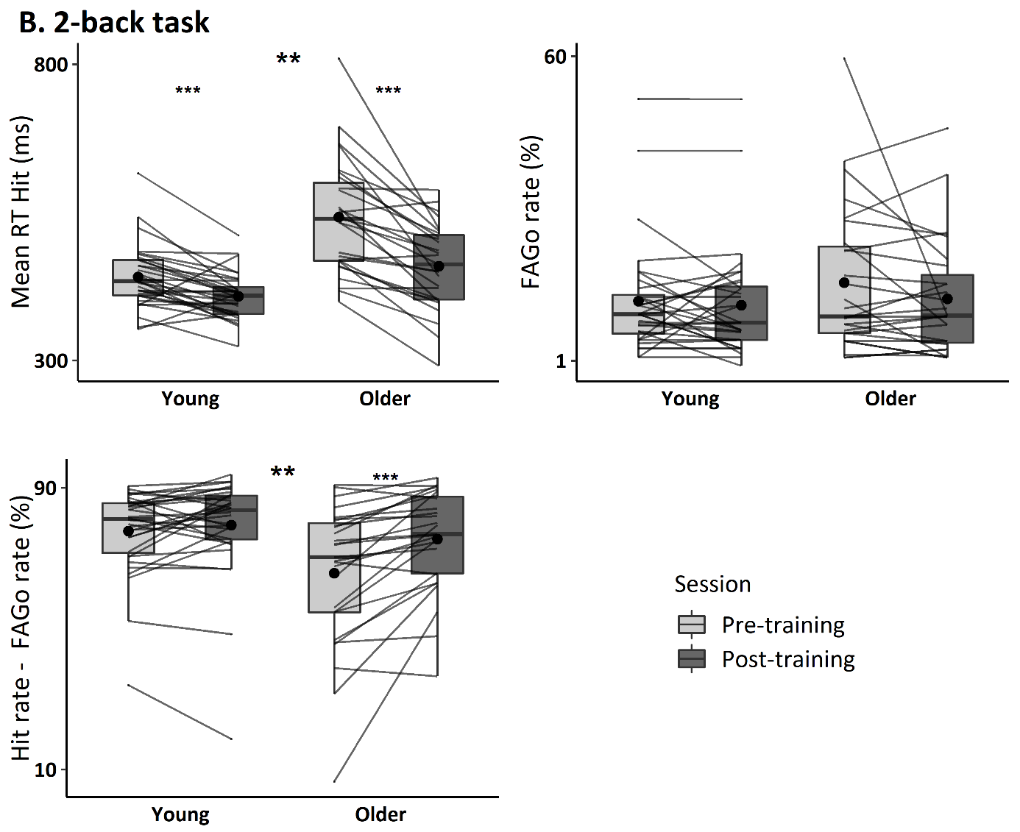


Supplementary figure 6. Behavioral performance during the 2-back task. Young and Older adults with Go/NoGo training are represented for all behavioral measures: Mean Response Time on Hit trials and False Alarm rate. Individual data points, means (bold circle), medians, first and third quartiles (horizontal bars), and the 1.5 inter-quartiles range (whiskers) are represented. *: p<.05, **: p<.01, ***: p<.001.

Omission rate at pre- and post-training during the Go/NoGo task

The omission rate (Miss rate) is defined as the rate of Go trials without motoric response.

## **Contrast 1: Session (Pre; Post-training) by Training (Go/NoGo training; Control 2-back training)**

There was no Session by Training interaction (F(1,55) = 0.15, p = .703, ηG² = .001), supported by a Bayes factors analysis (BF_01_ = 3.38).

**Supplementary table 5. Session by Training Miss rate at the Go/NoGo task**

|  | **Older Go/NoGo Training (n=29)** | | **Older Nback Training (n=28)** | |  |  |  |
| --- | --- | --- | --- | --- | --- | --- | --- |
| **Mean ±SD pre- post- t-test** | **Pre** | **Post** | **Pre** | **Post** | **Training Main Effect** | **Session Main Effect** | **Training x Session Interaction** |
| **Miss rate** | .02 ±.02 | .02 ±.02 | .02 ±.02 | .02 ±.02 | p = .684 η_G_² = .002 | p = .5 η_G_² = .003 | p = .703 η_G_² = .001 |
|  | p = .42 r = .15 | | p = .85 r = .04 | |  |  | BF01 = 3.38 |


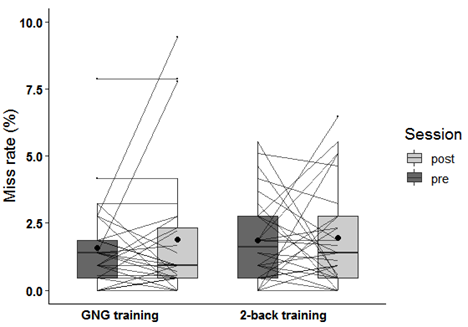


Supplementary figure 7. Miss rate during the Go/NoGo task. Older adults with Go/NoGo and 2-back training are represented for all behavioral measures: Mean Response Time on Hit trials and False Alarm rate. Individual data points, means (bold circle), medians, first and third quartiles (horizontal bars), and the 1.5 inter-quartiles range (whiskers) are represented. *: p<.05, **: p<.01, ***: p<.001.

## **Contrast 2: Session (Pre; Post-training) by Age (Young; Older adults)**

For the Miss rate, there was no Session by Age interaction (F(1,59) < 0.01, p = .998, η_G_² < .001), supported by a Bayes factors analysis (BF_01_ = 3.86).

**Supplementary table 6. Session by Age Miss rate at the Go/NoGo task**

|  | **Young**  **(n=32)** | | **Older Go/NoGo Training (n=29)** | |  |  |  |
| --- | --- | --- | --- | --- | --- | --- | --- |
| **Mean ±SD pre- post- t-test** | **Pre** | **Post** | **Pre** | **Post** | **Age**  **Main Effect** | **Session**  **Main Effect** | **Age x Session**  **Interaction** |
| **Miss rate** | .01 ±.02 | .01 ±.02 | .02 ±.02 | .02 ±.02 | p = .192  η_G_² = .022 | p = .213  η_G_² = .006 | p = .998  η_G_² < .001 |
|  | p = .34  r = .17 | | p = .42  r = .15 | |  |  | BF01 = 3.86 |


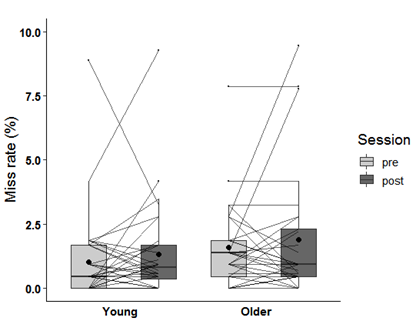


Supplementary figure 8. Miss rate during the Go/NoGo task. Young and Older adults are represented for all behavioral measures: Mean Response Time on Hit trials and False Alarm rate. Individual data points, means (bold circle), medians, first and third quartiles (horizontal bars), and the 1.5 inter-quartiles range (whiskers) are represented. *: p<.05, **: p<.01, ***: p<.001.

Electrical neuroimaging results comparison based on cueing condition

During the Go/NoGo task Pre- and Post-training sessions, trials were cued by a green or red circle, indicating a different probability of a Go trial to appear (cf main manuscript p10). To assess if the cueing condition had an influence, the neuroimaging analyses were redone on the red Cue trials only. The same procedure as for the main manuscript was followed for the pre-processing of the signal and for the analyses.

Supplementary table 7. Average number of Red Cue CR trials and rejected epochs for each group and both sessions

| **Mean ±SD** | **Young Go/NoGo training** | **Older Go/NoGo training** | **Older 2-back training** |
| --- | --- | --- | --- |
| **Red cueing CR trials** | 65.3 ±12.1 | 66.6 ±11.1 | 68.8 ±9.9 |
| **Rejected epochs** | 1.9 ±5.7 | 1.2 ±3.2 | 1.9 ±4.1 |

When taking only the red cueing trials, the neuroimaging results are identical to the results in the main manuscript for both Training by Session (supp. fig. 9) and Age by Session (supp. fig. 10) contrasts.


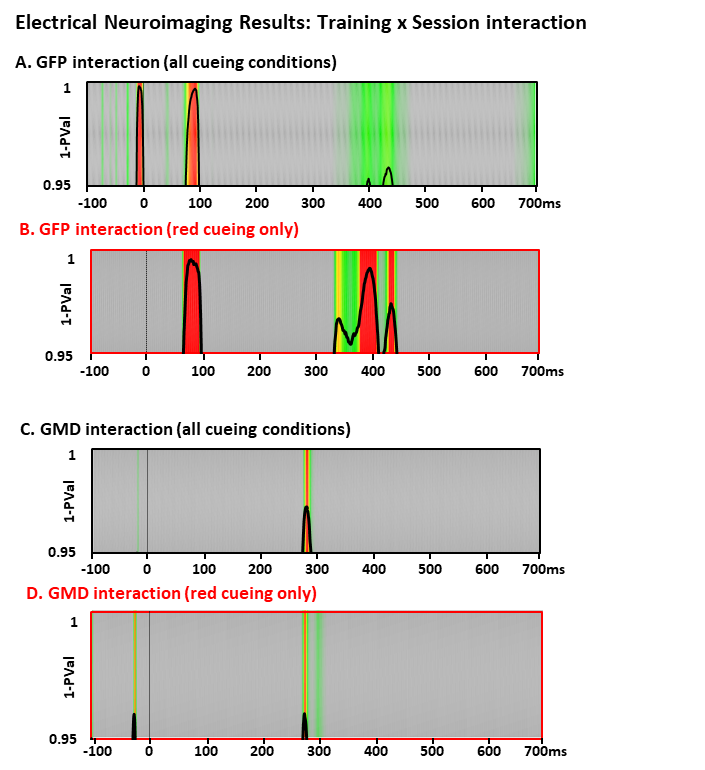


Supplementary figure. 9. Results of the global field power (A & B) and of the global map dissimilarity (C & D) Training by Session interaction revealed a sustained significant GFP but not topographic interaction during the P3 ERP component for both cueing conditions.


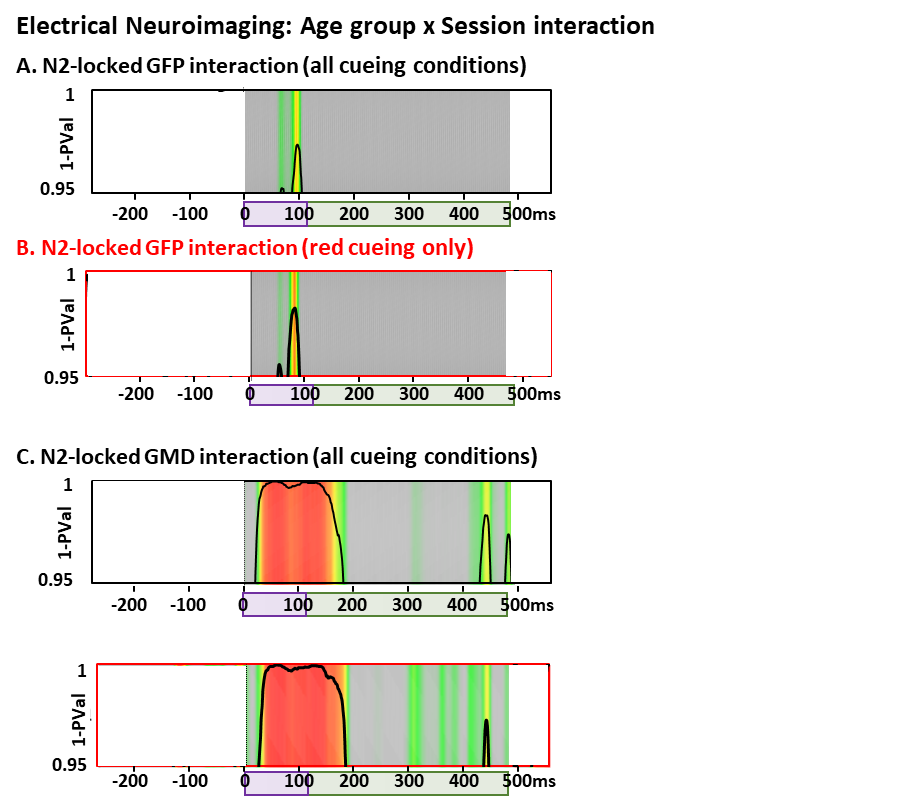


Supplementary figure. 10. Results of the global field power (A & B) and of the global map dissimilarity (C & D) Age by Session interaction revealed a sustained significant topographic but not GFP interaction during the N2 ERP component for both cueing conditions.

Response Time Threshold (RTT) at pre- and post-training during the Go/NoGo task

The RTT is an dynamic value determining if a response is too late or not. It was implemented in our task to ensure a fast response prepotency, and was continuously computed based on the median RT of the five precedent Hit trials (see main manuscript for more details).

Behavioral data pre-processing

For each participant, the final RTTs of all six Go/NoGo blocks were used to compute the participants’ median RTT.

## Contrast 1: **The effect of the Go/NoGo vs 2-Back training in older adults**

For the RTT, similar results to the RT Hit trials were observed (cf main manuscript; supp. table 9, supp. fig. 11). No interaction was found (F(55) < 0.01, p = .957, η_G_² < .001), supported by its Bayes Factor (BF01 = 3.54).

**Supplementary table 9. Session by Training RTT at the Go/NoGo task**

|  | **Older Go/NoGo Training**  **(n=29)** | | **Older Nback Training (n=28)** | |  |  |  |
| --- | --- | --- | --- | --- | --- | --- | --- |
| **Mean ±SD pre- post- t-test** | **Pre** | **Post** | **Pre** | **Post** | **Training**  **Main Effect** | **Session**  **Main Effect** | **Training x Session**  **Interaction** |
| **RTT** | 459 ±42 | 404 ±56 | 482 ±57 | 426 ±54 | p = .09  η_G_² = .046 | p < .001  η_G_² = .226 | p = .957  η_G_² < .001 |
|  | p < .001  r = .51 | | p < .001  r = .46 | |  |  | BF01 = 3.54 |


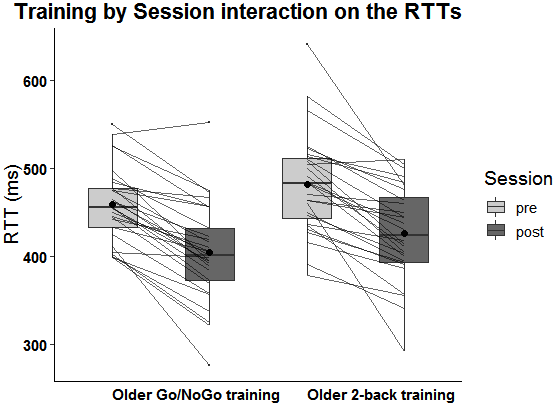


Supplementary figure 11. RTT during the Go/NoGo task. Older adults with Go/NoGo and 2-back training are represented for all behavioral measures: Mean Response Time on Hit trials and False Alarm rate. Individual data points, means (bold circle), medians, first and third quartiles (horizontal bars), and the 1.5 inter-quartiles range (whiskers) are represented. *: p<.05, **: p<.01, ***: p<.001.

## **Contrast 2: Session (Pre; Post-training) by Age (Young; Older adults)**

For the RTT, similar results to the RT Hit trials were observed (cf main manuscript; supp. table 10, supp. fig. 12). No interaction was found (F(59) = 2.06, p = .156, η_G_² = .004), but the absence of interaction is only lowly supported by its Bayes Factor (BF01 = 1.72).

**Supplementary table 10. Session by Age RTT at the Go/NoGo task**

|  | **Older Go/NoGo Training**  **(n=29)** | | **Young (n=32)** | |  |  |  |
| --- | --- | --- | --- | --- | --- | --- | --- |
| **Mean ±SD pre- post- t-test** | **Pre** | **Post** | **Pre** | **Post** | **Age**  **Main Effect** | **Session**  **Main Effect** | **Age x Session**  **Interaction** |
| **RTT** | 459 ±42 | 404 ±56 | 382.7 ±32 | 338 ±35 | p < .001  η_G_² = .426 | p < .001 η_G_² = .269 | p = .156  η_G_² = .004 |
|  | p < .001  r = .51 | | p < .001  r = .56 | |  |  | BF01 = 1.72 |


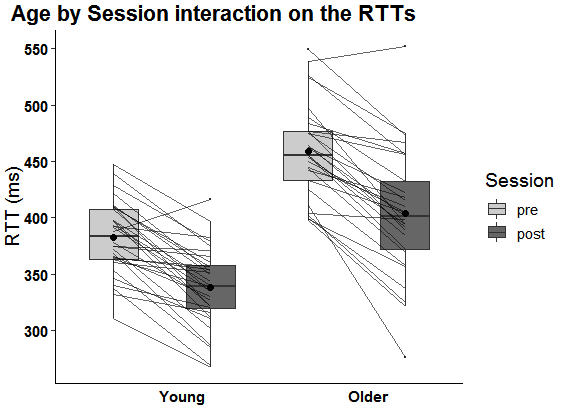


Supplementary figure 12. RTT during the Go/NoGo task. Young and Older adults are represented for all behavioral measures: Mean Response Time on Hit trials and False Alarm rate. Individual data points, means (bold circle), medians, first and third quartiles (horizontal bars), and the 1.5 inter-quartiles range (whiskers) are represented. *: p<.05, **: p<.01, ***: p<.001.
